# Analysis of the association between codon optimality and mRNA stability in *Schizosaccharomyces pombe*

**DOI:** 10.1186/s12864-016-3237-6

**Published:** 2016-11-08

**Authors:** Yuriko Harigaya, Roy Parker

**Affiliations:** 1 Department of Chemistry and Biochemistry, University of Colorado Boulder, Boulder, CO 80303 USA; 2 Howard Hughes Medical Institute, University of Colorado Boulder, Boulder, CO 80303 USA

**Keywords:** Codon optimality, mRNA stability, Evolutionary conservation

## Abstract

**Background:**

Recent experiments have shown that codon optimality is a major determinant of mRNA stability in *Saccharomyces cerevisiae* and that this phenomenon may be conserved in *Escherichia coli* and some metazoans, although work in *Neurospora crassa* is not consistent with this model.

**Results:**

We examined the association between codon optimality and mRNA stability in the fission yeast *Schizosaccharomyces pombe*. Our analysis revealed the following points. First, we observe a genome-wide association between codon optimality and mRNA stability also in *S. pombe*, suggesting evolutionary conservation of the phenomenon. Second, in both *S. pombe* and *S. cerevisiae*, mRNA synthesis rates are also correlated at the genome-wide analysis with codon optimality, suggesting that the long-appreciated association between codon optimality and mRNA abundance is due to regulation of both mRNA synthesis and degradation. However, when we examined correlation of codon optimality and either mRNA half-lives or synthesis rates controlling for mRNA abundance, codon optimality was still positively correlated with mRNA half-lives in *S. cerevisiae*, but the association was no longer significant for mRNA half-lives in *S. pombe* or for synthesis rates in either organism. This illustrates how only the pairwise analysis of multiple correlating variables may limit these types of analyses. Finally, in *S. pombe*, codon optimality is associated with known DNA/RNA sequence motifs that are associated with mRNA production/stability, suggesting these two features have been under similar selective pressures for optimal gene expression.

**Conclusions:**

Consistent with the emerging body of studies, this study suggests that the association between codon optimality and mRNA stability may be a broadly conserved phenomenon. It also suggests that the association can be explained at least in part by independent adaptations of codon optimality and other transcript features for elevated expression during evolution.

**Electronic supplementary material:**

The online version of this article (doi:10.1186/s12864-016-3237-6) contains supplementary material, which is available to authorized users.

## Background

The regulation of mRNA degradation is a critical step in the gene expression process. Previous studies have identified multiple gene sequence features that contribute to the dramatic differences in stability of cellular mRNAs. For example, RNA sequence elements [1, 2], RNA secondary structures within transcripts [3, 4], and DNA elements in promoter regions [5, 6], which can be specifically recognized by regulatory factors, play key roles in the modulation of mRNA stability [7]. Lengths of the transcripts can also affect mRNA stability [8, 9]. Moreover, a recent study by Coller and colleagues has identified codon optimality as another critical determinant of mRNA stability in *Saccharomyces cerevisiae* [10].

The concept of codon optimality has been developed through the study of the codon usage bias, which refers to the unequal frequency of synonymous codons within a gene, a genome or between genomes [11, 12]. The codon usage bias within a genome has been thought to be at least in part a consequence of selective pressure based on the observation that in multiple organisms highly expressed genes tend to have strong codon usage bias [13, 14]. Since the codons that are preferably used in highly expressed genes correspond to relatively abundant tRNA species in at least several organisms [15–17], it has been postulated that the preferred codons promote translation elongation rates and thereby increase the protein output. However, this hypothesis has been a matter of debate. A body of studies in unicellular organisms suggest that under physiological conditions rates of translation initiation rather than those of translation elongation mainly determine the protein output [18–21], which is not consistent with the hypothesis. On the other hand, some studies suggest that translation elongation rates can affect protein output by modulating translation initiation rates, which supports the hypothesis [22, 23].

The assumption underlying the hypothesis is that tRNA concentrations affect translation elongation rates at cognate codons. Because tRNA selection is a major rate-limiting step in the translation elongation cycle [24, 25], in theory, cellular concentrations of aminoacyl tRNAs affect rates of translation elongation when the cognate codons are in the ribosomal A-site. That is, the optimal codons, which are decoded by high abundance tRNA species, are translated more efficiently whereas the non-optimal codons, which are decoded by low abundance tRNA species, are translated more slowly. Despite the fundamental importance of this notion, it was only recently that convincing evidence emerged to support the idea that codon identify affects translation elongation rates [26–30].

In the recent study by Coller and colleagues, codon optimality was shown to be significantly associated with mRNA stability on a genomic scale in *S. cerevisiae* [10]. Moreover, experiments using several model transcripts have established a causal relationship between the two variables [10]. These results, along with earlier studies [31–33], have led to a model where non-optimal codons slow ribosomal translation and thereby trigger mRNA decay [10], which can offer a powerful explanation for the codon usage bias within a genome. More recent studies in *Escherichia coli*, zebrafish, *Drosophila*, *Xenopus*, and mouse are consistent with the codon-mediated regulation of mRNA stability being a conserved phenomenon [34–36]. Furthermore, the codon-mediated decay appears to play roles in human disease. For example, in human breast cancer cells, modulation of tRNA concentrations preferentially alters stability of mRNAs with high content of codons that are decoded by the tRNAs [37].

The generality of non-optimal codons triggering mRNA decay is not yet entirely clear. For example, a study in *Neurospora crassa* found that local accumulations of ribosomes on mRNAs due to synonymous codon substitutions, which indicate reduced rates of translation elongation, were not associated with changes in mRNA abundance [26]. The observation indicates that slow ribosome movements caused by non-optimal codons and mRNA instability can be uncoupled at least in certain situations and that the prevalence of the association between the two features requires additional investigation.

To examine the evolutionary conservation of the association between codon optimality and mRNA stability further, we have analyzed previously published RNA kinetic data in the fission yeast *Schizosaccharomyces pombe*, a yeast species that is evolutionarily distant from *S. cerevisiae* [38]. Our analysis revealed a significant association between codon optimality and mRNA stability in *S. pombe*, supporting the generality of the phenomenon. Our results also suggest that independent adaptations of codon optimality and other transcript features for elevated expression can contribute to formation of the optimal gene expression landscape.

## Results and discussion

### Comparison of previously published RNA kinetic datasets

As a prerequisite for our analysis to examine associations between codon optimality and mRNA stability, we required robust sets of genome-wide decay rate measurements. Towards this goal, we compiled previously published genome-wide RNA kinetic data in *S. cerevisiae* and *S. pombe*. The datasets were generated in multiple laboratories using various experimental methods and are summarized in Tables 1 and 2.

**Table 1:** Genome-wide RNA kinetic data in *S. cerevisiae*

Name	Reference	Method	Measured	Computed	Poly(A) enrichment	Quantification
Young	[52]	*rbp1-1*	Half-life	Synthesis rate	oligo(dT) selection	Microarray
Brown (1)	[50]	*rbp1-1*	Half-life	Synthesis rate^a^	None	Microarray
Brown (2)	[50]	*rbp1-1*	Half-life	Synthesis rate^a^	oligo(dT) priming	Microarray
Hughes	[53]	*rbp1-1*	Half-life	Synthesis rate^a^	oligo(dT) selection	Microarray
Peltz	[51]	*rbp1-1*	Half-life	Synthesis rate^a^	oligo(dT) selection	Microarray
Pilpel	[54]	*rbp1-1*	Half-life	Synthesis rate^a^	oligo(dT) priming	Microarray
Perez-Ortin	[77]	GRO	Synthesis rate	Half-life	None	Microarray
Cramer (1)	[44]	4sU labeling	Half-life	Synthesis rate	oligo(dT) priming	Microarray
Weis	[45]	4tU chase	Half-life	Synthesis rate^a^	oligo(dT) selection	RNA-seq
Cramer (2)	[48]	4tU labeling	Half-life	Synthesis rate	oligo(dT) priming	Microarray
Struhl	[8]	*rbp1-frb*	Half-life	Synthesis rate^a^	oligo(dT) priming	DRS
Gresham	[46]	4tU labeling	Half-life	Synthesis rate	None	RNA-seq
Coller (1)	[10]	*rbp1-1*	Half-life	Synthesis rate^a^	None	RNA-seq
Coller (2)	[10]	*rbp1-1*	Half-life	Synthesis rate^a^	oligo(dT) selection	RNA-seq

^a^The values were computed from mRNA half-lives in the source data and mRNA abundance obtained by Ito and colleagues [71]

**Table 2:** Genome-wide RNA kinetic data in *S. pombe*

Name	Reference	Method	Poly(A) enrichment	Quantification	Number of time points	Number of replicates	Labeling duration [min]
Mata (1)	[56]	4sU labeling	oligo(dT) priming	Microarray	1	1	15
Mata (2)	[56]	4sU labeling	oligo(dT) priming	Microarray	1	1	30
Mata (3)^a^	[47, 56]	4sU labeling	oligo(dT) priming	Microarray	1	1	15
Mata (4)^a^	[47, 56]	4sU labeling	oligo(dT) priming	Microarray	1	1	30
Cramer	[47]	4sU labeling	oligo(dT) priming	Microarray	1	2	6
Mata (5)	[57]	4sU labeling	oligo(dT) priming	Microarray	1	7	7 or 10
Gagneur	[58]	4tU labeling	None	RNA-seq	6	2	2, 4, 6, 8, 10, and steady state

^a^The “Mata (3)” and “Mata (4)” datasets contain values that were recomputed from the “Mata (1)” and “Mata (2)” data, respectively, taking into account labeling bias and cell division

In *S. cerevisiae*, a number of studies have measured mRNA synthesis and decay rates on a genomic scale. Although the methods used in these studies differ, kinetic rates are directly measured. Under the assumption that mRNA metabolism is in a steady state, some studies measured mRNA synthesis rates as well as mRNA abundance and calculated mRNA half-lives, while others measured mRNA decay rates and abundance to calculate mRNA synthesis rates (Table 1).

mRNA synthesis rates can be measured by multiple methods including genomic run-on (GRO) [39]. Direct measurement of mRNA half-lives is performed either by transcription shutoff or metabolic labeling. Although transcription shutoff is usually achieved by an addition of transcription inhibitor, in *S. cerevisiae*, many studies have used the *rpb1-1* temperature sensitive mutant of the *RBP1* gene, encoding the largest subunit of RNA polymerase II [40]. An alternative to the *rbp1-1* strain is the *rbp1-frb* strain, in which Rbp1 is depleted from the nucleus upon an addition of the drug rapamycin via the “anchor-away” technique [8, 41]. Metabolic labeling using 4-thiouridine (4sU) or 4-thiouracil (4tU) can measure mRNA half-lives with minimal perturbation [42–47]. In one scheme, time course experiments are performed after an addition of 4sU/4tU to cell cultures [44, 46–48], which we refer to as “4sU/4tU labeling.” In another scheme, samples are analyzed after an addition of Uracil to cultures that have been grown in the presence of 4sU/4tU [45], which we refer to as “4sU/4tU chase” (Table 1).

Genome-wide RNA kinetic measurement experiments also differ in methods of mRNA quantification, which can be performed by microarray, RNA-seq, and direct RNA sequencing (DRS). A critical factor in mRNA quantification is whether the procedure enriches polyadenylated species relative to deadenylated species for a given gene. The enrichment of polyadenylated species can be achieved by physical separation using an oligo(dT) column, which we refer to as “oligo(dT) selection.” It can also be achieved by selective cDNA synthesis using an oligo(dT) primer, which we refer to as “oligo(dT) priming” (Table 1). It was previously noted that oligo(dT) selection can fail to capture RNA species that retain substantial poly(A) tails and result in highly skewed kinetic values [10, 49].

**Fig. 1:**
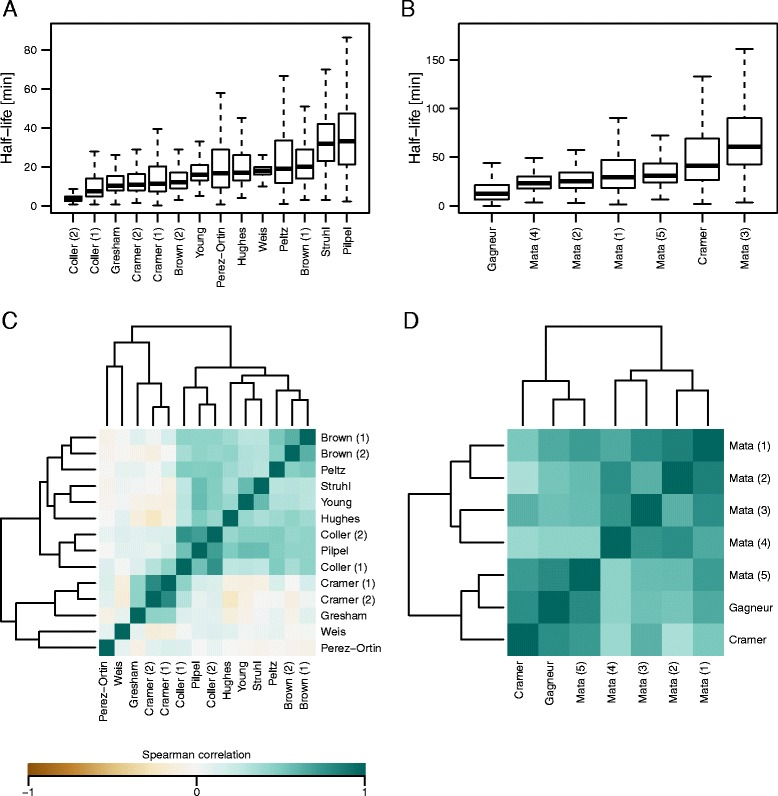
Comparison of mRNA half-life measurements in *S. cerevisiae* and *S. pombe.* **a** Boxplots of mRNA half-lives from 14 datasets in *S. cerevisiae*. The datasets are ordered by median values. **b** Boxplots of mRNA half-lives from seven datasets in *S. pombe*. **c** Heatmap showing pairwise Spearman correlation coefficients of mRNA half-life measurements in *S. cerevisiae*. The datasets are clustered via hierarchical clustering based on Euclidian distances. **d** Heatmap showing pairwise Spearman correlation coefficients of mRNA half-life measurements in *S. pombe*

**Fig. 2:**
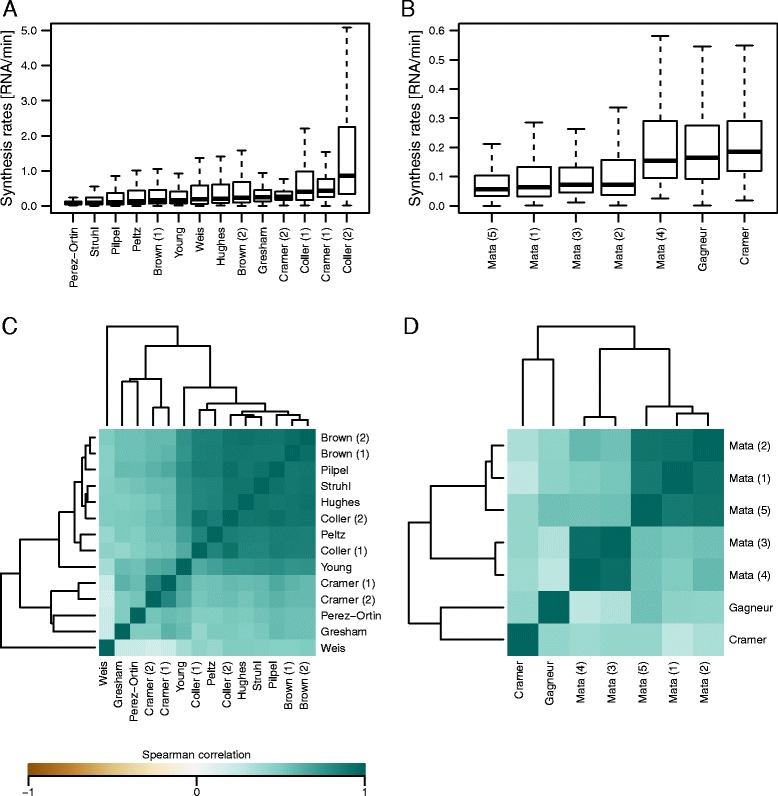
Comparison of mRNA synthesis rates in *S. cerevisiae* and *S. pombe.* **a** Boxplots of mRNA synthesis rates from 14 datasets in *S. cerevisiae*. The datasets are ordered by median values. **b** Boxplots of mRNA synthesis rates from seven datasets in *S. pombe*. **c** Heatmap showing pairwise Spearman correlation coefficients of mRNA synthesis rates in *S. cerevisiae*. The datasets are clustered via hierarchical clustering based on Euclidian distances. **d** Heatmap showing pairwise Spearman correlation coefficients of mRNA synthesis rates in *S. pombe*

We first compared mRNA half-lives and synthesis rates obtained from 14 previously published RNA kinetic data in *S. cerevisiae* (Methods and Table 1). The analysis led to the following observations. First, as previously noted by others [10, 44, 47], ranges of mRNA half-lives varied substantially depending on the dataset (Fig. 1a), and correlations across different datasets were generally poor (Fig. 1c and Additional file 1: Figure S1). Second, however, datasets that were generated via 4sU/4tU labeling protocols (“Cramer (1),” “Cramer (2),” and “Gresham”) [44, 46, 48] showed similar ranges of values (Fig. 1a) and fair levels of correlations (Fig. 1c and Additional file 1: Figure S1). Although datasets that were generated via the *rbp1-1* or *rbp1-frb* system (“Brown (1),” “Brown (2),” “Peltz,” “Struhl,” “Young,” “Hughes,” “Coller (2),” “Pilpel,” and “Coller (1)”) [8, 10, 50–54] also showed fair levels of correlations (Fig. 1c), their absolute values varied substantially (Fig. 1a). We note that mRNA half-lives obtained by Weis and colleagues via 4tU chase and oligo(dT) selection (“Weis”) [45] substantially differed from the 4sU/4tU labeling data (“Cramer (1),” “Cramer (2),” and “Gresham”), which were generated via protocols without oligo(dT) selection (Table 1, Fig. 1c, and Additional file 1: Figure S1). Third, unlike mRNA half-lives, mRNA synthesis rates from all datasets were positively correlated with one another regardless of the methods used for measurements (Fig. 2c and Additional file 2: Figure S3). We note, however, that the absolute values differed substantially depending on the datasets (Fig. 2a).

Although it is not possible to rigorously determine which datasets are most biologically accurate, we suggest that the results obtained via 4sU/4tU labeling without oligo(dT) selection (“Cramer (1)”, “Cramer (2),” and “Gresham”) are more likely to reflect the physiological nature of gene regulation for the following reasons. First, methods that involve transcription shutoff are subject to artifacts due to stress responses caused by global transcription shutoff [47]. Second, in the GRO protocols, cells are treated with sarkosyl, which perturbs cell physiology. Third, at least in *S. cerevisiae*, it has been shown that an addition of 4sU/4tU at a relevant concentration does not impact gene expression profiles within a relevant time frame [44, 47] and that an addition of 4tU at a relevant concentration does not noticeably affect cell growth [46]. Fourth, as described above, oligo(dT) selection can lead to skewed values [10]. We, therefore, focused on the three datasets (“Cramer (1),” “Cramer (2),” and “Gresham”) in subsequent analyses in *S. cerevisiae*.

We also compared mRNA half-life values in seven datasets from four studies in *S. pombe* (“Mata (1),” “Mata (2),” “Mata (3),” “Mata (4),” “Cramer,” “Mata (5),” and “Gagneur”), which were all generated via 4sU/4tU labeling (Methods and Table 2). In addition, we also analyzed genome-wide chromatin immunoprecipitation (ChIP) data for RNA polymerase II (RNAPII) by Bahler and colleagues (“Bahler”) [55] because an association of RNAPII can serve as a proxy for mRNA synthesis. The analysis led to the following observations. First, mRNA half-lives in the different datasets showed moderate to strong correlations with one another (Fig. 1d and Additional file 3: Figure S2). Second, mRNA synthesis rates were also well correlated across the datasets (Fig. 2d, Additional file 4: Figure S4, and Additional file 5: Figure S5). Third, however, the absolute values of mRNA half-lives and synthesis rates differed substantially depending on the datasets (Figs. 1b and 2b). These observations represent the technical challenges in quantifying absolute kinetic values but suggest that relative values are reasonably reliable.

Although all these datasets were generated via 4sU/4tU labeling, the quality of data is likely to be heterogeneous (Table 2). For example, the two datasets obtained from an early study by Mata and colleagues (“Mata (1)” and “Mata (2)”) do not contain replicates [56]. Moreover, in the experiments, cells were labeled with 4sU for 15 and 30 min [56], which could potentially have affected cell physiology. The same caveats apply to the “Mata (3)” and “Mata (4)” datasets, which contain values recomputed by Cramer and colleagues from the “Mata (1)” and “Mata (2)” datasets, respectively, taking into account labeling bias and cell division [47]. The dataset by Cramer and colleagues (“Cramer”) were obtained from two replicate experiments in which cells were labeled with 4sU for 6 min [47]. The more recent data by Mata and colleagues (“Mata (5)”) contain arithmetic means of values obtained from seven replicate experiments in which cells were labeled with 4sU for either 7 or 10 min [57]. The most recent data by Gagneur, Cramer, and colleagues (“Gagneur”) are based on two replicate experiments in which samples were analyzed at five time points within 10 min after an addition of 4tU as well as at the steady state. Moreover, unlike in other studies, the kinetic rates were obtained by a mathematical model that takes into account pre-mRNA splicing and, therefore, are likely to be more accurate [58]. To control the quality, in subsequent analyses, we focused on the two datasets obtained from the most recent studies, which consist of larger numbers of replicates/data points (“Mata (5)” and “Gagneur”).

### Correlations between mRNA half-lives, synthesis rates, and abundance

We next examined relationships between mRNA half-lives, stability, and abundance in the selected datasets, which we considered the most reliable. The analysis led to the following observations, which are consistent with previous studies [44, 55]. First, mRNA half-lives were positively correlated with mRNA abundance in both *S. cerevisiae* (Additional file 6: Figures S6A-C) and *S. pombe* (Additional file 7: Figures S7A and B). Second, mRNA synthesis rates were also positively correlated with mRNA levels in both organisms (Additional file 6: Figures S6D-F, Additional file 7: Figure S7C, and D). Third, mRNA half-lives and synthesis rates did not show a consistent relationship across datasets in *S. cerevisiae* (Additional file 6: Figure S6G-I) whereas, in *S. pombe*, very weak positive correlations were detected (Additional file 7: Figure S7G and H).

Overall, the results are consistent with a global picture of gene regulation in dividing cells of *S. cerevisiae* and *S. pombe*, where both mRNA synthesis rates and half-lives are positively correlated with mRNA abundance. Rather than being a fine tuner, mRNA stability control appears to function cooperatively with the transcriptional control to contribute to the diversification of mRNA abundance.

### Association between codon optimality and mRNA stability

To examine whether codon optimality is associated with mRNA stability in *S. pombe*, we used a method previously developed by Coller and colleagues with a minor modification (Methods) [10]. As a metric of optimality of each codon, we used both the “relative adaptiveness value” for the tRNA adaptation index (tAI) (Methods) [59], also known as classical translation efficiency (cTE) [60], and normalized translation efficiency (nTE) [60]. tAI takes into account tRNA gene copy numbers and wobble base paring based on the assumption that tRNA gene copy numbers correspond to aminoacyl tRNA concentrations. nTE additionally takes into account mRNA abundance based on the idea that an effective tRNA concentration can also be affected by the levels of mRNAs that require the tRNA for translation.

We first calculated a metric called the codon occurrence to mRNA stability correlation coefficient (CSC), which was originally defined as a Pearson's correlation coefficient between the frequency of occurrence of each codon in mRNAs and mRNA half-lives [10]. In our analysis, we used a Spearman's correlation coefficient instead of a Pearson's correlation coefficient since some data contained outliers. Codons that are enriched in stable mRNAs have positive CSC values, whereas those that are enriched in unstable mRNA have negative CSC values. To determine the statistical significance of the association between codon optimality and the CSC, we split codons into two groups based on the sign of the CSC and performed chi-square tests as in the previous study [10].

**Fig. 3:**
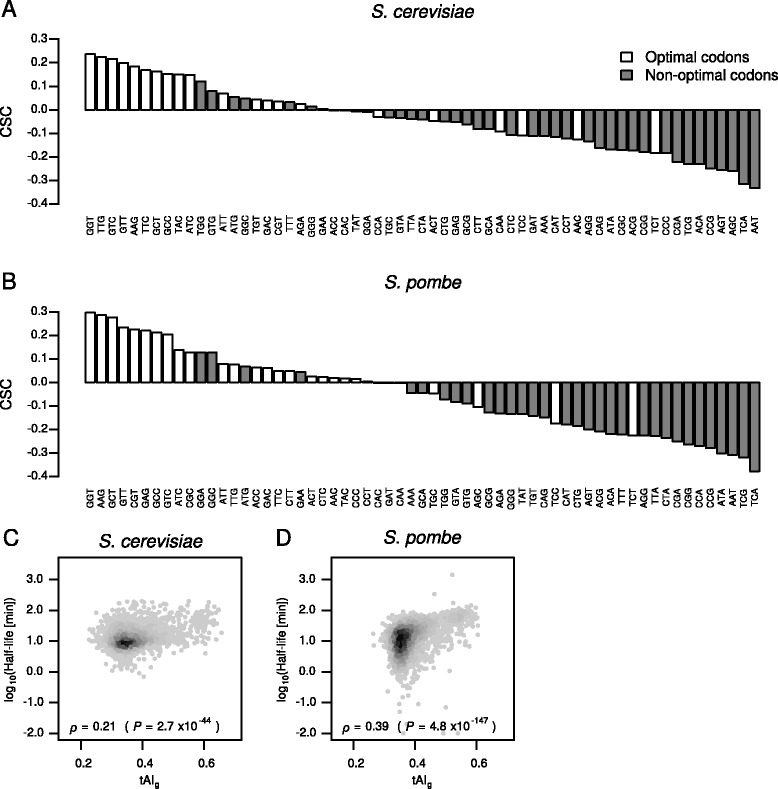
Codon optimality and mRNA half-lives are significantly associated in *S. cerevisiae* and *S. pombe*. **a** The CSC plotted for each codon based on *S. cerevisiae* mRNA half-lives in the “Gresham” dataset. The *white* and *gray bars* represent optimal and non-optimal codons, respectively. The classification of codon optimality is based on the *S. cerevisiae* cTE. **b** The CSC plotted for each codon based on *S. pombe* mRNA half-lives in the “Gagneur” dataset. The classification of codon optimality is based on the *S. pombe* cTE. **c** Scatterplot comparing tAI_g_ and mRNA half-lives in the “Gresham” dataset in *S. cerevisiae*. Spearman's *ρ* and *P* value are shown. **d** Scatterplot comparing tAI_g_ and mRNA half-lives in the “Gagneur” dataset in *S. pombe*

Coller and colleagues have observed the association between codon optimality and mRNA half-lives in their own data generated via the *rbp1-1* system (“Coller (1)”) as well as in the data by Cramer and colleagues, which was generated via 4sU labeling (“Cramer (1)”) [10, 44]. To reassess these results in *S. cerevisiae*, we computed the CSC values using the dataset by Coller and colleagues (“Coller (1)”) as well as those on which we chose to focus in this study (“Cramer (1),” “Cramer (2),” and “Gresham”). This analysis led to the following observations, which confirmed the previously discovered association in *S. cerevisiae*. First, CSC values obtained from the four datasets were highly correlated with one another (Additional file 8: Figure S8A-F). Second, the CSC values were significantly associated with the both metrics of codon optimality (cTE and nTE) (Fig. 3a, Additional file 9: Figure S9A, B, and Table 3) [10]. Third, consistent with this, mRNA half-lives were positively correlated with geometric mean values of tAI computed for individual genes (tAI_g_) (Methods) [59] (Fig. 3c, Additional file 9: Figure S9D, and E). Moreover, we obtained essentially the same results when we used percent optimal codon content of individual genes instead of the tAI_g_ values (Additional file 10: Figure S10A-F). Fourth, the CSC values were positively correlated with the tAI values (Additional file 11: Figure S11A, D, and G). Fifth, similarly to the earlier study [10], the positive correlation between codon optimality and the CSC disappeared when +1 or +2 frameshifts were computationally introduced, suggesting that other transcript features, such as nucleotide composition and RNA structures, are unlikely to contribute to the observed correlation (Additional file 11: Figure S11B, C, E, F, H, and I).

**Table 3:** Association between codon optimality and mRNA half-lives

	cTE				nTE			
	Total	Observed	Expected	*P* value	Total	Observed	Expected	*P* value
*S. cerevisiae*								
Cramer (1)	25	23	9.8	1.5e-11	25	21	12.3	1.9e-05
Cramer (2)	26	18	10.2	1.2e-04	26	19	12.8	3.1e-03
Gresham	22	16	8.7	1.9e-04	22	17	10.8	2.4e-03
*S. pombe*								
Mata (5)	27	24	12.4	9.2e-09	27	22	15.5	1.7e-03
Gagneur	26	22	11.9	6.7e-07	26	21	14.9	3.5e-03

Total numbers of codons with positive CSC values (“Total”) among 61 codons, observed numbers of optimal codons with positive CSC values (“Observed”), expected numbers of optimal codons with positive CSC values (“Expected”), and *P* values from chi-square tests

We then performed similar analyses using the two RNA kinetic datasets selected in *S. pombe* (“Mata (5)” and “Gagneur”). This led to the following observations. First, the CSC values obtained from the two datasets were highly correlated with each other (Additional file 8: Figure S8G). Second, the CSC values were significantly associated with the both metrics of codon optimality (cTE and nTE) (Fig. 3b, Additional file 9: Figure S9C, and Table 3). Third, consistent with this, mRNA half-lives were positively correlated with the tAI_g_ values as well as with optimal codon content (Fig. 3d, Additional file 9: Figure S9F, and Additional file 10: Figure S10G-J). Fourth, the CSC values were positively correlated with the tAI values (Additional file 12: Figure S12A and D). Fifth, the positive correlation between codon optimality and the CSC disappeared when +1 or +2 frameshifts were computationally introduced (Additional file 12: Figure S12B, C, E, and F).

These results suggest that the association between codon optimality and mRNA stability is conserved when examined at a gnome-wide scale in *S. pombe*. Given the evolutionary distance between the two yeast species [38], this implies that the association is a broadly conserved phenomenon.

Although we are only presenting a correlation between codon optimality and decay rates, several observations suggest that codon optimality can impact the rate of mRNA decay through a mechanistic connection, at least in *S. cerevisiae*. First, in the replacement of abundant codons with minor synonymous codons in the *S. cerevisiae PGK1* transcript reduced the mRNA level, consistent with reduced codon optimality increasing the decay rate [31]. Second, resynthesis of mRNAs with increased or decreased codon optimality but synonymous codons leads to a corresponding change in the mRNA decay rate [10]. Third, specific regions of minor synonymous codons can lead to increased mRNA decay rates for the *S. cerevisiae MATalpha1* mRNA [32, 33]. An important issue for future work will be determining the mechanistic coupling between codon optimality and mRNA decay mechanisms. Notably, new studies have started elucidating the molecular mechanisms underlying the coupling [35, 36, 61].

It has long been appreciated that highly expressed genes are often enriched in optimal codons [11]. To investigate the association between codon optimality and mRNA half-lives further, we computed the Spearman's partial correlations between the two variables controlling for mRNA abundance. In *S. cerevisiae*, the positive correlation between codon optimality in all three metrics and mRNA half-lives remained significant after analyses were controlled for mRNA abundance (Additional file 13: Table S1). This is consistent with the intimate relationship between codon optimality and mRNA stability in this organism. In *S. pombe*, the positive correlation remained for both the datasets only when the tAI values were used as a metric of codon optimality (Additional file 13: Table S1). This may suggest that, in *S. pombe*, the relationship between codon optimality and mRNA stability is weaker than in *S. cerevisiae*.

### Association between codon optimality and mRNA synthesis rates

**Fig. 4:**
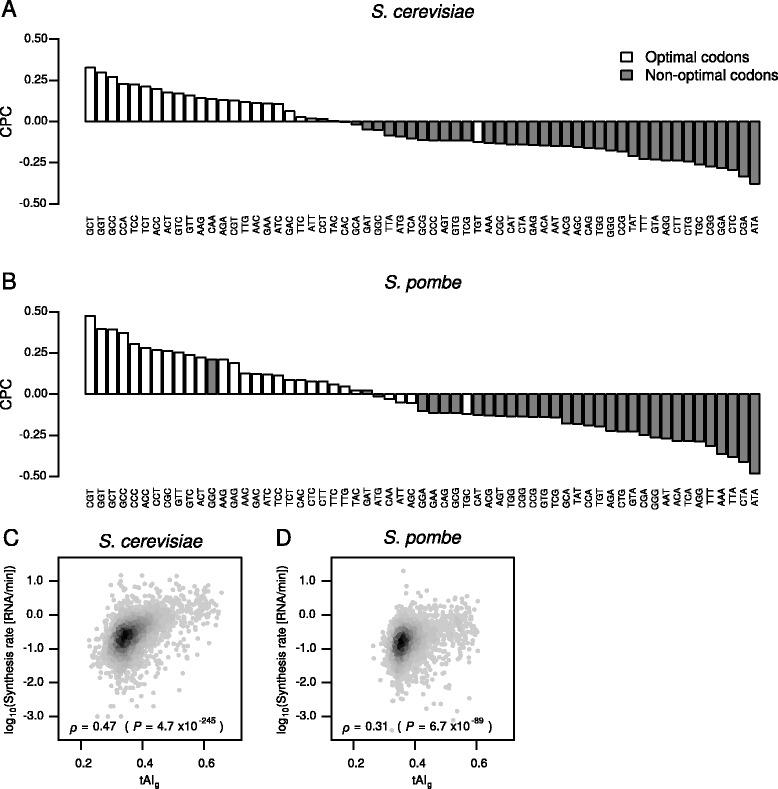
Codon optimality and mRNA synthesis rates are significantly associated in *S. cerevisiae* and *S. pombe*. **a** The CPC plotted for each codon based on *S. cerevisiae* mRNA synthesis rates in the “Gresham” dataset. The *white* and *gray bars* represent optimal and non-optimal codons, respectively. The classification of codon optimality is based on the *S. cerevisiae* cTE. **b** The CPC plotted for each codon based on *S. pombe* mRNA synthesis rates in the “Gagneur” dataset. The classification of codon optimality is based on the *S. pombe* cTE. **c** Scatterplot comparing tAI_g_ and mRNA synthesis rates in the “Gresham” dataset in *S. cerevisiae*. Spearman's *ρ* and *P* value are shown. **d** Scatterplot comparing tAI_g_ and mRNA synthesis rates in the “Gagneur” dataset in *S. pombe*

As described above, highly expressed genes are often enriched in optimal codons [11]. Since mRNA abundance is determined by rates of mRNA production and degradation, we next examined the association between codon optimality and mRNA synthesis rates in *S. cerevisiae* and *S. pombe*. For this purpose, we calculated a Spearman's correlation coefficient between the frequency of occurrence of each codon in mRNAs and mRNA synthesis rates, which we termed the codon occurrence to mRNA production correlation coefficient (CPC). In *S. pombe*, we also calculated the CPC values based on the genome-wide ChIP data for RNAPII by Bahler and colleagues [55]. The analysis led to the following observations. First, CPC values obtained from all the analyzed datasets were highly correlated (Additional file 14: Figure S13). Second, the CPC values were significantly associated with tAI (cTE) as well as with nTE in both *S. cerevisiae* and *S. pombe* (Fig. 4a, b, Additional file 15: Figure S14A, B, Additional file 16: Figure S15A, B, and Table 4). Third, mRNA synthesis rates were positively correlated with the tAI_g_ values as well as optimal codon content (Additional file 15: Figures S14C, D, Additional file 16: Figure S15C, D, and Additional file 17: Figure S16). Fourth, the CPC values were positively correlated with the tAI values (Additional file 18: Figure S17A, D, G, Additional file 19: Figure S18A, D, and G). Fifth, the positive correlation between codon optimality and the CPC disappeared when +1 or +2 frameshifts were computationally introduced [10] (Additional file 18: Figure S17B, C, E, F, H, I, Additional file 19: Figure S18B, C, E, F, H, and I).

**Table 4:** Association between codon optimality and mRNA synthesis rates

	cTE				nTE			
	Total	Observed	Expected	*P* value	Total	Observed	Expected	*P* value
*S. cerevisiae*								
Cramer (1)	23	23	9.0	3.5e-13	23	20	11.3	1.5e-05
Cramer (2)	22	17	8.7	1.9e-05	22	16	10.8	1.3e-02
Gresham	23	22	9.0	1.7e-11	23	19	11.3	1.5e-04
*S. pombe*								
Bahler	27	25	12.4	3.8e-10	27	23	15.5	2.6e-04
Mata (5)	21	18	9.6	2.1e-05	21	18	12.0	3.0e-03
Gagneur	26	24	11.9	1.9e-09	26	22	14.9	5.7e-04

Total numbers of codons with positive CPC values (“Total”) among 61 codons, observed numbers of optimal codons with positive CPC values (“Observed”), expected numbers of optimal codons with positive CPC values (“Expected”), and *P* values from chi-square tests

These results suggest that, in both *S. cerevisiae* and *S. pombe*, not only mRNA half-lives but also mRNA synthesis rates are correlated with codon optimality and that the long-appreciated association between codon optimality and mRNA abundance is due to regulation of both mRNA synthesis and degradation. The association between codon optimality and mRNA synthesis rates would be most readily explained by independent adaptations of codon usage and other gene features that modulate mRNA synthesis rates for elevated expression during evolution.

To investigate the association between codon optimality and mRNA synthesis rates in more detail, we computed the Spearman's partial correlations between the two variables controlling for mRNA abundance (Additional file 20: Table S2). With one exception in which we performed the analysis using optimal codon content in the nTE classification and the “Mata (5)” dataset, we found no significant positive partial correlations between codon optimality and mRNA synthesis rates once we controlled for mRNA abundance. This can be consistent with the relationship between codon optimality and mRNA synthesis rates being indirect in both organisms.

### Association between codon optimality and DNA/RNA sequence motifs

The above analysis revealed a consistent correlation between aspects of mRNAs that promote gene expression. *Cis*-acting DNA/RNA sequence motifs are also critical determinants in the regulation of mRNA production and stability. It is possible that the association between codon optimality and mRNA production/stability is in part due to associations between codon optimality and such sequence elements. In *S. cerevisiae*, to our knowledge, identification of sequence elements that are associated with mRNA synthesis rates and/or half-lives based on 4sU/4tU labeling data has not been performed. Moreover, our attempt to identify such sequence motifs was not successful. Consistent with this, a recent study suggests that inherent transcript properties including codon content rather than specific sequence motifs account for a large fraction of variation in mRNA decay rates in this organism [62]. On the other hand, in *S. pombe*, Gagneur, Cramer, and colleagues have identified DNA/RNA sequence motifs that are associated with mRNA synthesis rates and/or half-lives based on their 4tU labeling data [58].

**Fig. 5:**
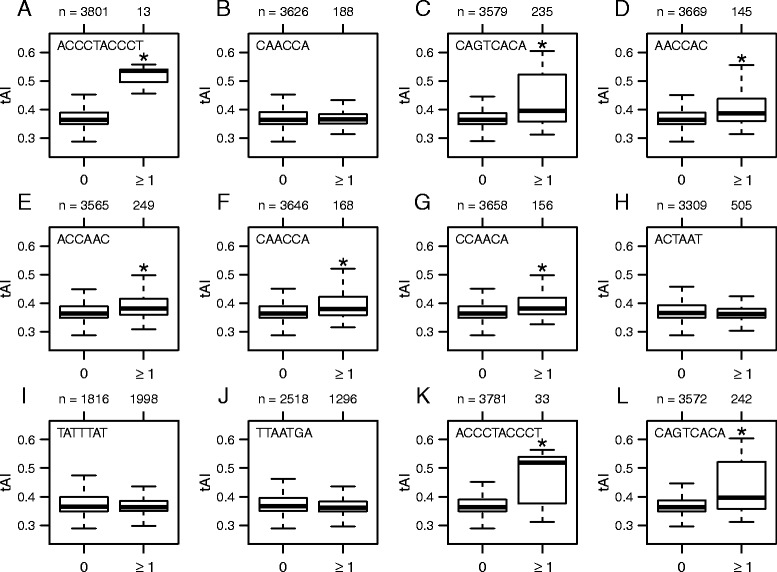
Associations between codon optimality and DNA/RNA motifs. **a**–**l** Boxplots comparing codon optimality (tAI_g_) between genes containing known motifs that are associated with mRNA synthesis rates and/or half-lives (≥1) and those lacking them (0). The asterisk indicates that the tAI_g_ values of the former group of genes (≥1) are significantly greater than those of the latter group (0) (Bonferroni-corrected Wilcoxon rank-sum test *P* < 0.05). See also Table 5

To address the question of whether the sequence elements and codon optimality have been under similar selective pressures for optimal gene expression, we compared the tAI_g_ values between genes containing the previously identified motifs [58] and those lacking them in *S. pombe*. Interestingly, we observed that the tAI_g_ values of genes with motifs that were located in promoter and/or 5' UTR regions and associated with long half-lives and/or fast synthesis rates are significantly greater than those of genes without them (Fig. 5a, c-g, k, l, and Table 5). On the other hand, there was no significant difference in the tAI_g_ values between genes containing motifs that are associated with short half-lives and those lacking them (Fig. 5b, h, i, and Table 5) with one exception of the TTAATGA motif located in 3' UTR (Fig. 5j and Table 5). Essentially the same results were obtained when optimal codon content was compared instead of the tAI_g_ values (Table 5).

**Table 5:** Association between codon optimality and DNA/RNA sequence motifs

			tAI_g_		Percent optimal codons (cTE)		Percent optimal codons (nTE)	
Motif	Location	Rate	Greater	Less	Greater	Less	Greater	Less
ACCCTACCCT	Promoter	Long half-life	8.6e-07	1	4.2e-07	1	2.7e-06	1
CAACCA	Promoter	Short half-life	1	1	1	1	1	1
CAGTCACA	Promoter	Long half-life	7.2e-19	1	6.7e-18	1	3.4e-18	1
AACCAC	5' UTR	Long half-life	7.6e-10	1	3.3e-10	1	8.0e-10	1
ACCAAC	5' UTR	Long half-life	4.5e-12	1	4.5e-12	1	2.4e-10	1
CAACCA	5' UTR	Long half-life	3.0e-07	1	3.6e-10	1	7.5e-08	1
CCAACA	5' UTR	Long half-life	1.1e-08	1	2.3e-08	1	1.2e-07	1
ACTAAT	3' UTR	Short half-life	1	0.05	1	0.23	1	1
TATTTAT	3' UTR	Short half-life	1	0.03	1	0.52	1	1
TTAATGA	3' UTR	Short half-life	1	6.8e-06	1	1.1e-05	1	7.1e-03
ACCCTACCCT	Promoter	Fast synthesis	2.1e-07	1	1.1e-07	1	2.1e-07	1
CAGTCACA	Promoter	Fast synthesis	1.4e-18	1	5.9e-18	1	4.6E-18	1

Bonferroni-corrected *P* values from Wilcoxon rank sum tests comparing the tAI_g_ values or optimal codon content of genes containing the motifs and those lacking them. The “greater” and “less” indicate the alternative hypotheses that the tAI_g_ values or optimal codon content of the former group of genes are greater and less than those of the latter, respectively

These results are consistent with the idea that codon usage and certain DNA/RNA sequence motifs have been under similar selective pressures. Interestingly, the 5' UTR elements are enriched near the 5' end of mRNA not near the start codon [58] and, therefore, are unlikely to define the start codon context, which is already known to be associated with codon optimality in certain organisms as well as with mRNA stability in *S. pombe* [21, 55, 63, 64]. Rather, the motifs may modulate 5 ' cap accessibility, which is associated with translation initiation efficiency [29, 65]. Because of the causal relationship between efficient translation initiation and mRNA stability [66, 67], these observations collectively suggest that the association between codon optimality and mRNA stability is at least in part attributable to similar selective pressures acting on codon optimality and 5' UTR sequence features that may modulate translation initiation efficiency.

## Conclusion

Our study in *S. cerevisiae* and *S. pombe* suggests that the genome-wide association between codon optimality and mRNA stability is a conserved phenomenon. It also suggests that the association in *S. pombe* is at least in part attributable to independent adaptations of codon usage and other transcript features during evolution. Although this study did not find such transcript features in *S. cerevisiae*, it cannot be excluded that independent adaptations contribute to the association also in this organism. As has been performed in *S. cerevisiae*, it will be important to experimentally examine causality between the two variables in *S. pombe*. Determining relative contributions from direct mechanistic links and selective pressures will be a key step to a fuller understanding of mRNA stability control.

## Methods

### Data sources

Coding sequences and annotations of *S. cerevisiae* (version R64-1-1) and *S. pombe* (version asm294v2.26) were obtained from the Saccharomyces genome database [68], Pombase [69], and Ensembl Genomes [70]. Sources of RNA kinetic data are summarized in Tables 1 and 2. mRNA abundance data were taken from previous studies by Ito and colleagues [71], Cramer and colleagues [44, 48], Gresham and colleagues [46], and Bahler and colleagues [72]. The relative adaptiveness values for tRNA adaptation index (tAI) were taken from a previous study by Tuller and colleagues [73]. The gene-wise average tAI values (tAI_g_) were computed using the codonR program developed by dos Reis and colleagues [59]. Classification of optimal and non-optimal codons was taken from a previous study by Frydman and colleagues [60]. Genome-wide ChIP data for RNAPII in *S. pombe* was taken from a previous study by Bahler and colleagues [55].

### Data filtering and processing

Out of all 6717 annotated ORFs in *S. cerevisiae*, we included all 4789 nuclear-encoded ORFs that are annotated as “verified” (Additional file 21: Table S3) [68]. Out of all 5142 annotated ORFs in *S. pombe*, we included all nuclear-encoded ORFs except those designated as dubious and SPAC1556.06, which has multiple transcript isoforms [69]. This resulted in 5051 genes (Additional file 22: Table S4). From the RNA kinetic data by Gagneur, Cramer, and colleagues [58], we excluded multicistronic transcript units. We used “minute,” “molecule per minute per cell,” and “molecule per cell” as units of mRNA half-lives, synthesis rates, and abundance, respectively. When the values were expressed otherwise in the source data, we converted them accordingly. In our calculations, we used a total number of mRNAs per cell of 60,000 and that of 41,000 for *S. cerevisiae* and *S. pombe*, respectively [72, 74]. When mRNA synthesis rates were not available in the source data, based on the steady-state assumption [75], we computed them from mRNA half-lives and abundance using the equations *λ* = log2/*hl* and *μ* = *m* (*α* + *λ*), where *λ* is the mRNA decay rate [min^−1^], *hl* is the mRNA half-life [min], *μ* is the mRNA synthesis rate [RNA min^−1^ cell^−1^], *m* is the mRNA abundance [cell^−1^], and *α* is the cell growth rate [min^−1^]. When mRNA abundance was not available in the source data, we used data obtained by Ito and colleagues [71] and those obtained by Bahler and colleagues [72] for *S. cerevisiae* and *S. pombe*, respectively. For the datasets in *S. cerevisiae*, we used mRNA abundance obtained in matched types of culture media. That is, we used mRNA abundance in the rich nutrient YPD medium for the “Brown (1),” “Brown (2),” “Hughes,” “Peltz,” “Pilpel,” “Struhl,” “Coller (1),” and “Coller (2)” datasets, whereas we used that in the minimal nutrient SC medium for the “Weis” dataset (Additional file 21: Table S3). For mRNA half-live data obtained via the *rbp1-1* and *rbp1-frb* systems, where cell growth is halted, or those obtained without taking into account cell division, we set *α* to zero. For the “Weis” data, we computed *α* from a cell cycle length of 150 min using the equation *α* = log2/*ccl*, where ccl is the cell cycle length [min].

### Statistical analyses and graphics

All statistical analyses were performed using R [76]. The chisq.test() function was used to perform chi-square tests. The cor.test() function was used to calculate Spearman's correlation coefficients. The wilcox.test() function was used to perform Wilcoxon rank sum tests. The pcor.test() function in the ppcor package was used to calculate Spearman's partial correlations. The heatscatter() function in the LSD package was used to draw scatterplots. The heatmap.2() function in the gplots package was modified to draw heatmaps.

### Calculation of CSC and CPC

We calculated the CSC as described previously except that we used Spearman's correlation coefficients instead of Pearson's correlation coefficients [10]. We calculated the CPC by replacing mRNA half-lives with mRNA synthesis rates in the CSC calculation.

### Motif analysis

The DNA/RNA motifs were taken from a previous study by Gagneur, Cramer, and colleagues [58]. The motifs TATTTAT, TATTTA and ATTTAT were combined into a group (denoted as TATTTAT). The motifs TTAATGA, TTAATG, and TAATGA were also combined (denoted as TTAATGA). Only significant motif instances were considered (*P* < 0.05). As a background gene set, we considered 3814 genes for which mRNA half-lives and synthesis rates were estimated. Information about the presence and absence of the motifs can be found in Additional file 23: Table S5.

## Additional files

Additional file 1: Figure S1. Comparison of mRNA half-life measurements in *S. cerevisiae*. The pairwise scatterplots compare mRNA half-lives in 14 datasets. The datasets are ordered as in Fig. 1c. The upper triangle panels show Spearman correlation coefficients (top) and *P* values (bottom). The axis range is from 0 to 60 min. (PDF 1243 kb)
Additional file 2: Figure S3. Comparison of mRNA synthesis rates in *S. cerevisiae*. The pairwise scatterplots compare mRNA synthesis rates in 14 datasets. The datasets are ordered as in Fig. 2c. The upper triangle panels show Spearman correlation coefficients (top) and *P* values (bottom). The axis range is from 0 to 1 RNA per minute. (PDF 1203 kb)
Additional file 3: Figure S2. Comparison of mRNA half-life measurements in *S. pombe*. The pairwise scatterplots compare mRNA half-lives in seven datasets. The datasets are ordered as in Fig. 1d. The upper triangle panels show Spearman correlation coefficients (top) and *P* values (bottom). The axis range is from 0 to 120 min. (PDF 456 kb)
Additional file 4: Figure S4. Comparison of mRNA synthesis rates in *S. pombe*. The pairwise scatterplots compare mRNA half-lives in seven datasets. The datasets are ordered as in Fig. 2d. The upper triangle panels show Spearman correlation coefficients (top) and *P* values (bottom). The axis range is from 0 to 1 RNA per minute. (PDF 434 kb)
Additional file 5: Figure S5. Comparison of mRNA synthesis rates with ChIP intensity signals for RNAPII in *S. pombe*. (A-G) Scatterplot comparing mRNA synthesis rates [RNA/min] in seven datasets and RNAPII ChIP signals in an arbitrary unit (a. u.) obtained by Bahler and colleagues [55]. Spearman's *ρ* and *P* value are shown. (PDF 984 kb)
Additional file 6: Figure S6. Correlations between mRNA half-lives, synthesis rates, and abundance in *S. cerevisiae*. (A) Scatterplot comparing mRNA half-lives and abundance in the “Cramer (1)” data. Spearman's *ρ* and *P* value are shown. (B) Same as (A) but for the “Cramer (2)” data. (C) Same as (A) but for the “Gresham” data. (D) Scatterplot comparing mRNA synthesis rates and abundance in the “Cramer (1)” data. (E) Same as (D) but for the “Cramer (2)” data. (F) Same as (D) but for the “Gresham” data. (G) Scatterplot comparing mRNA half-lives and synthesis rates in the “Cramer (1)” data. (H) Same as (G) but for the “Cramer (2)” data. (I) Same as (G) but for the “Gresham” data. (PDF 1187 kb)
Additional file 7: Figure S7. Correlations between mRNA half-lives, synthesis rates, and abundance in *S. pombe*. (A) Scatterplot comparing mRNA half-lives in the “Mata (5)” data and mRNA abundance [RNA/cell] obtained by Bahler and colleagues [72]. Spearman's *ρ* and *P* value are shown. (B) Same as (A) but for mRNA half-lives in the “Gagneur” data. (C) Scatterplot comparing mRNA synthesis rates computed from the “Mata (5)” data and mRNA abundance as shown in (A). (D) Same as (C) but for mRNA synthesis rates in the “Gagneur” data. (E) Scatterplot comparing mRNA half-lives and synthesis rates in the “Mata (5)” data. (F) Same as (E) but for the “Gagneur” data. (PDF 818 kb)
Additional file 8: Figure S8. Correlations between the CSC values obtained from different RNA kinetic datasets in *S. cerevisiae* (“Cramer (1),” “Cramer (2),” “Gresham,” and “Coller (1)”) (A-F) and *S. pombe* (“Mata (5)” and “Gagneur”) (G). Spearman's *ρ* and *P* value are shown. The circles and cross signs represent optimal and non-optimal codons, respectively. (PDF 38 kb)
Additional file 9: Figure S9. Codon optimality and mRNA half-lives are significantly associated in *S. cerevisiae* and *S. pombe*. (A) The CSC plotted for each codon based on *S. cerevisiae* mRNA half-lives in the “Cramer (1)” dataset. The white and gray bars represent optimal and non-optimal codons, respectively. The classification of codon optimality is based on the *S. cerevisiae* cTE. (B) Same as (A) but for the “Cramer (2)” dataset. (C) The CSC plotted for each codon based on *S. pombe* mRNA half-lives in the “Mata (5)” dataset. The classification of codon optimality is based on the *S. pombe* cTE. (D) Scatterplot comparing tAI_g_ and mRNA half-lives in the “Cramer (1)” dataset in *S. cerevisiae*. Spearman's *ρ* and *P* value are shown. (E) Same as (D) but for the “Cramer (2)” data. (F) Same as (D) but for the “Mata (5)” dataset in *S. pombe*. (PDF 427 kb)
Additional file 10: Figure S10. Correlations between optimal codon content and mRNA half-lives in *S. cerevisiae* and *S. pombe*. (A) Scatterplot comparing optimal codon content based on the cTE classification and mRNA half-lives in the “Cramer (1)” data in *S. cerevisiae*. Spearman's *ρ* and *P* value are shown. (B) Same as (A) but for the “Cramer (2)” data. (C) Same as (A) but for the “Gresham” data. (D) Same as (A) but based on the nTE classification. (E) Same as (D) but for the “Cramer (2)” data. (F) Same as (D) but for the “Gresham” data. (G) Same as (A) but for the “Mata (5)” data in *S. pombe*. (H) Same as (G) but for the “Gagneur” data. (I) Same as (G) but based on the nTE classification. (J) Same as (I) but for the “Gagneur” data. (PDF 1341 kb)
Additional file 11: Figure S11. Introduction of +1 and +2 frameshifts eliminates the positive correlation between codon optimality and the CSC in *S. cerevisiae*. (A) Scatterplot comparing the tAI values and the CSC based on the “Cramer (1)” data (*ρ* = 0.76, *P* = 1.7 × 10^−12^). The circles and cross signs represent optimal and non-optimal codons, respectively. (B) Same as (A) but upon introduction of +1 frameshifts (*ρ* = −0.12, *P* = 0.37). (C) Same as (A) but upon introduction of +2 frameshifts (*ρ* = −0.34, *P* = 7.7 × 10^−3^). (D) Same as (A) but based on the “Cramer (2)” data (*ρ* = 0.62, *P* = 9.3 × 10^−8^). (E) Same as (B) but based on the “Cramer (2)” data (*ρ* = −0.16, *P* = 0.21). (F) Same as (C) but based on the “Cramer (2)” data (*ρ* = −0.29, *P* = 0.02). (G) Same as (A) but based on the “Gresham” data (*ρ* = 0.58, *P* = 1.0 × 10^−6^). (H) Same as (B) but based on the “Gresham” data (*ρ* = 0.03, *P* = 0.76). (I) Same as (C) but based on the “Gresham” data (*ρ* = −0.23, *P* = 0.07). (PDF 29 kb)
Additional file 12: Figure S12. Introduction of +1 and +2 frameshifts eliminates the positive correlation between codon optimality and the CSC in *S. pombe*. (A) Scatterplot comparing the tAI values and the CSC based on the “Mata (5)” data (*ρ* = 0.85, *P* = 7.6 × 10^−18^). The circles and cross signs represent optimal and non-optimal codons, respectively. (B) Same as (A) but upon introduction of +1 frameshifts (*ρ* = −0.16, *P* = 0.22). (C) Same as (A) but upon introduction of +2 frameshifts (*ρ* = −0.30, *P* = 0.02). (D) Same as (A) but based on the “Gagneur” data (*ρ* = 0.81, *P* = 4.6 × 10^−15^). (E) Same as (B) but based on the “Gagneur” data (*ρ* = −0.16, *P* = 0.02). (F) Same as (C) but based on the “Gagneur” data (*ρ* = −0.37, *P* = 3.8 × 10^−3^). (PDF 24 kb)
Additional file 13: Table S1. Spearman's correlation coefficients and Spearman's partial correlations controlling for mRNA abundance to assess an association between codon optimality and mRNA half-lives. (XLS 31 kb)
Additional file 14: Figure S13. Correlations between the CPC values obtained from different RNA kinetic datasets in *S. cerevisiae* (“Cramer (1),” “Cramer (2),” and “Gresham”) (A-C) and *S. pombe* (“Mata (5)” and “Gagneur”) as well as those obtained from the RNAPII ChIP data in *S. pombe* (“Bahler”) (D-F) [55]. Spearman's *ρ* and *P* value are shown. The circles and cross signs represent optimal and non-optimal codons, respectively. (PDF 36 kb)
Additional file 15: Figure S14. Codon optimality and mRNA synthesis rates are significantly associated in *S. cerevisiae*. (A) The CPC plotted for each codon based on mRNA synthesis rates in the “Cramer (1)” dataset. The white and gray bars represent optimal and non-optimal codons, respectively. The classification of codon optimality is based on the *S. cerevisiae* cTE. (B) Same as (A) but based on the “Cramer (2)” dataset. (C) Scatterplot comparing tAI_g_ and mRNA synthesis rates in the “Cramer (1)” dataset. Spearman's *ρ* and *P* value are shown. (D) Same as (C) but for the “Cramer (2)” dataset. (PDF 275 kb)
Additional file 16: Figure S15. Codon optimality and mRNA synthesis rates are significantly associated in *S. pombe*. (A) The CPC plotted for each codon based on the RNAPII ChIP signals by Bahler and colleagues [55]. The white and gray bars represent optimal and non-optimal codons, respectively. The classification of codon optimality is based on the *S. pombe* cTE. (B) Same as (A) but for the CPC based on mRNA synthesis rates in the “Mata (5)” dataset. (C) Scatterplot comparing tAI_g_ and RNAPII ChIP signals by Bahler and colleagues. Spearman's *ρ* and *P* value are shown. (D) Same as (C) but for mRNA synthesis rates in the “Mata (5)” dataset. (PDF 323 kb)
Additional file 17: Figure S16. Correlations between optimal codon content and mRNA synthesis rates in *S. cerevisiae* and *S. pombe*. (A) Scatterplot comparing optimal codon content based on the cTE classification and mRNA synthesis rates in the “Cramer (1)” data in *S. cerevisiae*. Spearman's *ρ* and *P* value are shown. (B) Same as (A) but for the “Cramer (2)” data. (C) Same as (A) but for the “Gresham” data. (D) Same as (A) but based on the nTE classification. (E) Same as (D) but for the “Cramer (2)” data. (F) Same as (D) but for the “Gresham” data. (G) Same as (A) but for the *S. pombe* RNAPII ChIP data by Bahler and colleagues. (H) Same as (A) but for the “Mata (5)” data in *S. pombe*. (I) Same as (G) but for the “Gagneur” data. (J) Same as (G) but based on the nTE classification. (K) Same as (J) but for the “Mata (5)” data. (L) Same as (J) but for the “Gagneur” data. (PDF 1653 kb)
Additional file 18: Figure S17. Introduction of +1 and +2 frameshifts eliminates the positive correlation between codon optimality and the CPC in *S. cerevisiae*. (A) Scatterplot comparing the tAI values and the CPC based on the “Cramer (1)” data (*ρ* = 0.79, *P* = 6.6 × 10^−14^). The circles and cross signs represent optimal and non-optimal codons, respectively. (B) Same as (A) but upon introduction of +1 frameshifts (*ρ* = −0.10, *P* = 0.44). (C) Same as (A) but upon introduction of +2 frameshifts (*ρ* = −0.28, *P* = 0.03). (D) Same as (A) but based on the “Cramer (2)” data (*ρ* = 0.74, *P* = 1.4 × 10^−11^). (E) Same as (B) but based on the “Cramer (2)” data (*ρ* = −0.16, *P* = 0.22). (F) Same as C but based on the “Cramer (2)” data (*ρ* = −0.05, *P* = 0.72). (G) Same as (A) but based on the “Gresham” data (*ρ* = 0.75, *P* = 3.4 × 10^−12^). (H) Same as (B) but based on the “Gresham” data (*ρ* = −0.17, *P* = 0.18). (I) Same as (C) but based on the “Gresham” data (*ρ* = −0.15, *P* = 0.26). (PDF 29 kb)
Additional file 19: Figure S18. Introduction of +1 and +2 frameshifts eliminates the positive correlation between codon optimality and the CPC in *S. pombe*. (A) Scatterplot comparing the tAI values and the CPC based on the RNAPII ChIP signals by Bahler and colleagues (*ρ* = 0.80, *P* = 5.5 × 10^−15^). The circles and cross signs represent optimal and non-optimal codons, respectively. (B) Same as (A) but upon introduction of +1 frameshifts (*ρ* = −0.14, *P* = 0.29). (C) Same as (A) but upon introduction of +2 frameshifts (*ρ* = −0.22, *P* = 0.09). (D) Same as (A) but for the CPC based on mRNA synthesis rates in the “Mata (5)” data (*ρ* = 0.83, *P* = 1.4 × 10^−16^). (E) Same as (B) but for the CPC based on mRNA synthesis rates in the “Mata (5)” data (*ρ* = −0.15, *P* = 0.25). (F) Same as (C) but for the CPC based on mRNA synthesis rates in the “Mata (5)” data (*ρ* = −0.19, *P* = 0.13). (G) Same as (D) but based on the “Gagneur” data (*ρ* = 0.82, *P* = 4.2 × 10^−16^). (H) Same as (E) but based on the “Gagneur” data (*ρ* = −0.20, *P* = 0.11). (I) Same as (F) but based on the “Gagneur” data (*ρ* = −0.28, *P* = 0.03). (PDF 29 kb)
Additional file 20: Table S2. Spearman's correlation coefficients and Spearman's partial correlations controlling for mRNA abundance to assess an association between codon optimality and mRNA synthesis rates. (XLS 31 kb)
Additional file 21: Table S3. tAI_g_ values, percent optimal codons, mRNA half-lives synthesis rates, and abundance in *S. cerevisiae*. (XLS 3062 kb)
Additional file 22: Table S4. tAI_g_ values, percent optimal codons, mRNA half-lives, synthesis rates, and abundance in *S. pombe*. (XLS 2000 kb)
Additional file 23: Table S5. Presence (1) and absence (0) of DNA/RNA sequence motifs in *S. pombe*. (XLS 538 kb)

## Abbreviations

3' UTR: Three prime untranslated region
4sU: 4-thiouridine
4tU: 4-thiouracil
5' UTR: Five prime untranslated region
ChIP: Chromatin immunoprecipitation
CPC: Codon occurrence to mRNA production correlation coefficient
CSC: Codon occurrence to mRNA stability correlation coefficient
cTE: Classical translation efficiency
DRS: Direct RNA sequencing
GRO: Genomic run-on
nTE: Normalized translation efficiency
RNAPII: RNA polymerase II
tAI: tRNA adaptation index
