# Supplementary material for: Analysis of the association between codon optimality and mRNA stability in Schizosaccharomyces pombe
Source: BMC Genomics. 2016 Nov 8;17:895. doi: 10.1186/s12864-016-3237-6 (PMC5101800; doi:10.1186/s12864-016-3237-6)
Supplement: Additional file 1: Figure S1. — Comparison of mRNA half-life measurements in S. cerevisiae. The pairwise scatterplots compare mRNA half-lives in 14 datasets. The datasets are ordered as in Fig. 1c. The upper triangle panels show Spearman correlation coefficients (top) and P values (bottom). The axis range is from 0 to 60 min. (PDF 1243 kb) [file 12864_2016_3237_MOESM1_ESM.pdf]

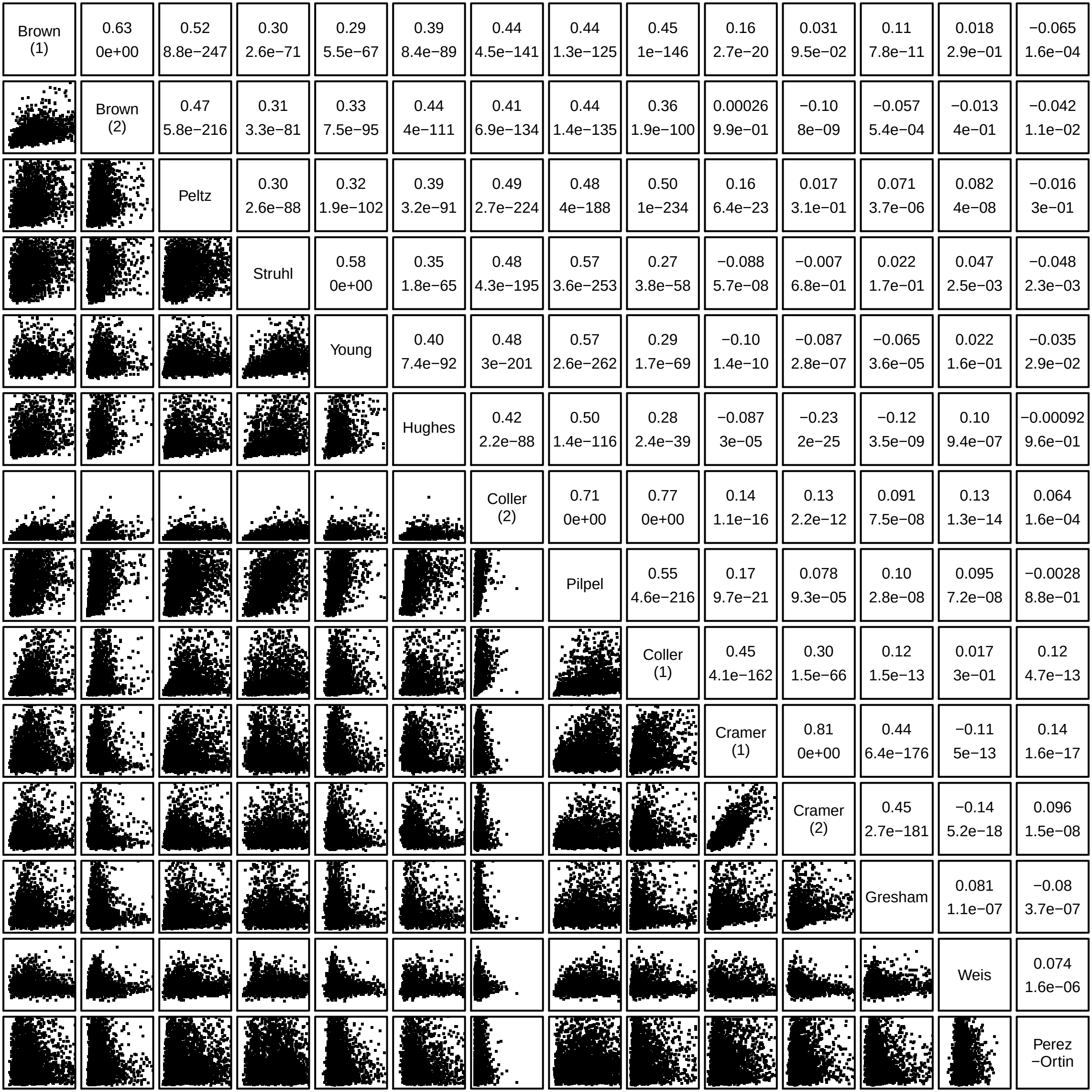

Brown (1)
0.63 0e+00
0.52 8.8e−247
0.30 2.6e−71
0.29 5.5e−67
0.39 8.4e−89
0.44 4.5e−141
0.44 1.3e−125
0.45 1e−146
0.16 2.7e−20
0.031 9.5e−02
0.11 7.8e−11
0.018 2.9e−01
−0.065 1.6e−04
Brown (2)
0.47 5.8e−216
0.31 3.3e−81
0.33 7.5e−95
0.44 4e−111
0.41 6.9e−134
0.44 1.4e−135
0.36 1.9e−100
0.00026 9.9e−01
−0.10 8e−09
−0.057 5.4e−04
−0.013 4e−01
−0.042 1.1e−02
Peltz
0.30 2.6e−88
0.32 1.9e−102
0.39 3.2e−91
0.49 2.7e−224
0.48 4e−188
0.50 1e−234
0.16 6.4e−23
0.017 3.1e−01
0.071 3.7e−06
0.082 4e−08
−0.016 3e−01
Struhl
0.58 0e+00
0.35 1.8e−65
0.48 4.3e−195
0.57 3.6e−253
0.27 3.8e−58
−0.088 5.7e−08
−0.007 6.8e−01
0.022 1.7e−01
0.047 2.5e−03
−0.048 2.3e−03
Young
0.40 7.4e−92
0.48 3e−201
0.57 2.6e−262
0.29 1.7e−69
−0.10 1.4e−10
−0.087 2.8e−07
−0.065 3.6e−05
0.022 1.6e−01
−0.035 2.9e−02
Hughes
0.42 2.2e−88
0.50 1.4e−116
0.28 2.4e−39
−0.087 3e−05
−0.23 2e−25
−0.12 3.5e−09
0.10 9.4e−07
−0.00092 9.6e−01
Coller (2)
0.71 0e+00
0.77 0e+00
0.14 1.1e−16
0.13 2.2e−12
0.091 7.5e−08
0.13 1.3e−14
0.064 1.6e−04
Pilpel
0.55 4.6e−216
0.17 9.7e−21
0.078 9.3e−05
0.10 2.8e−08
0.095 7.2e−08
−0.0028 8.8e−01
Coller (1)
0.45 4.1e−162
0.30 1.5e−66
0.12 1.5e−13
0.017 3e−01
0.12 4.7e−13
Cramer (1)
0.81 0e+00
0.44 6.4e−176
−0.11 5e−13
0.14 1.6e−17
Cramer (2)
0.45 2.7e−181
−0.14 5.2e−18
0.096 1.5e−08
Gresham
0.081 1.1e−07
−0.08 3.7e−07
Weis
0.074 1.6e−06
Perez −Ortin
