# Supplementary material for: Analysis of the association between codon optimality and mRNA stability in Schizosaccharomyces pombe
Source: BMC Genomics. 2016 Nov 8;17:895. doi: 10.1186/s12864-016-3237-6 (PMC5101800; doi:10.1186/s12864-016-3237-6)
Supplement: Additional file 2: Figure S3. — Comparison of mRNA synthesis rates in S. cerevisiae. The pairwise scatterplots compare mRNA synthesis rates in 14 datasets. The datasets are ordered as in Fig. 2c. The upper triangle panels show Spearman correlation coefficients (top) and P values (bottom). The axis range is from 0 to 1 RNA per minute. (PDF 1203 kb) [file 12864_2016_3237_MOESM2_ESM.pdf]

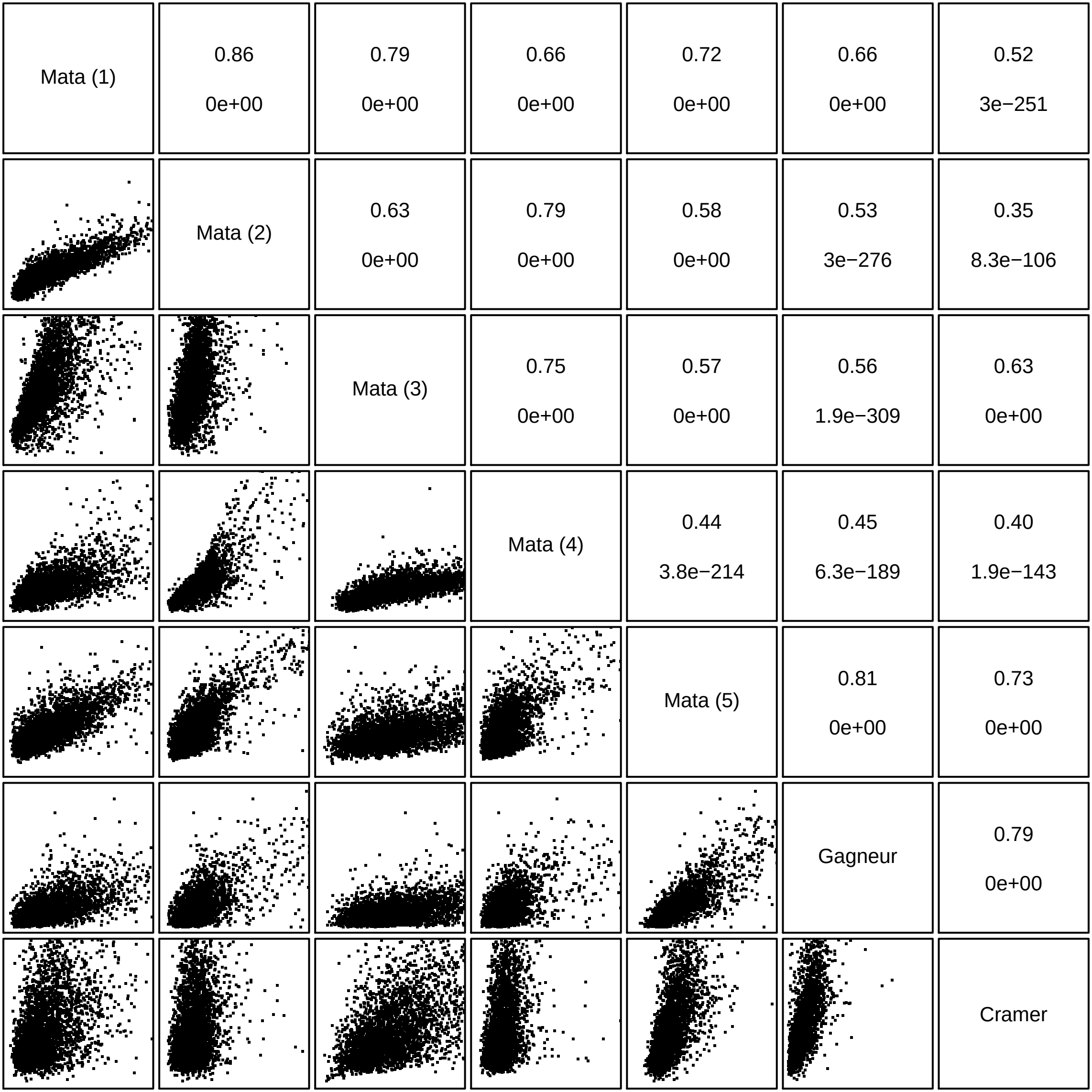
Mata (1)
0.86
0e+00
0.79
0e+00
0.66
0e+00
0.72
0e+00
0.66
0e+00
0.52
3e−251
Mata (2)
0.63
0e+00
0.79
0e+00
0.58
0e+00
0.53
3e−276
0.35
8.3e−106
Mata (3)
0.75
0e+00
0.57
0e+00
0.56
1.9e−309
0.63
0e+00
Mata (4)
0.44
3.8e−214
0.45
6.3e−189
0.40
1.9e−143
Mata (5)
0.81
0e+00
0.73
0e+00
Gagneur
0.79
0e+00
Cramer
