# Supplementary material for: Analysis of the association between codon optimality and mRNA stability in Schizosaccharomyces pombe
Source: BMC Genomics. 2016 Nov 8;17:895. doi: 10.1186/s12864-016-3237-6 (PMC5101800; doi:10.1186/s12864-016-3237-6)
Supplement: Additional file 3: Figure S2. — Comparison of mRNA half-life measurements in S. pombe. The pairwise scatterplots compare mRNA half-lives in seven datasets. The datasets are ordered as in Fig. 1d. The upper triangle panels show Spearman correlation coefficients (top) and P values (bottom). The axis range is from 0 to 120 min. (PDF 456 kb) [file 12864_2016_3237_MOESM3_ESM.pdf]

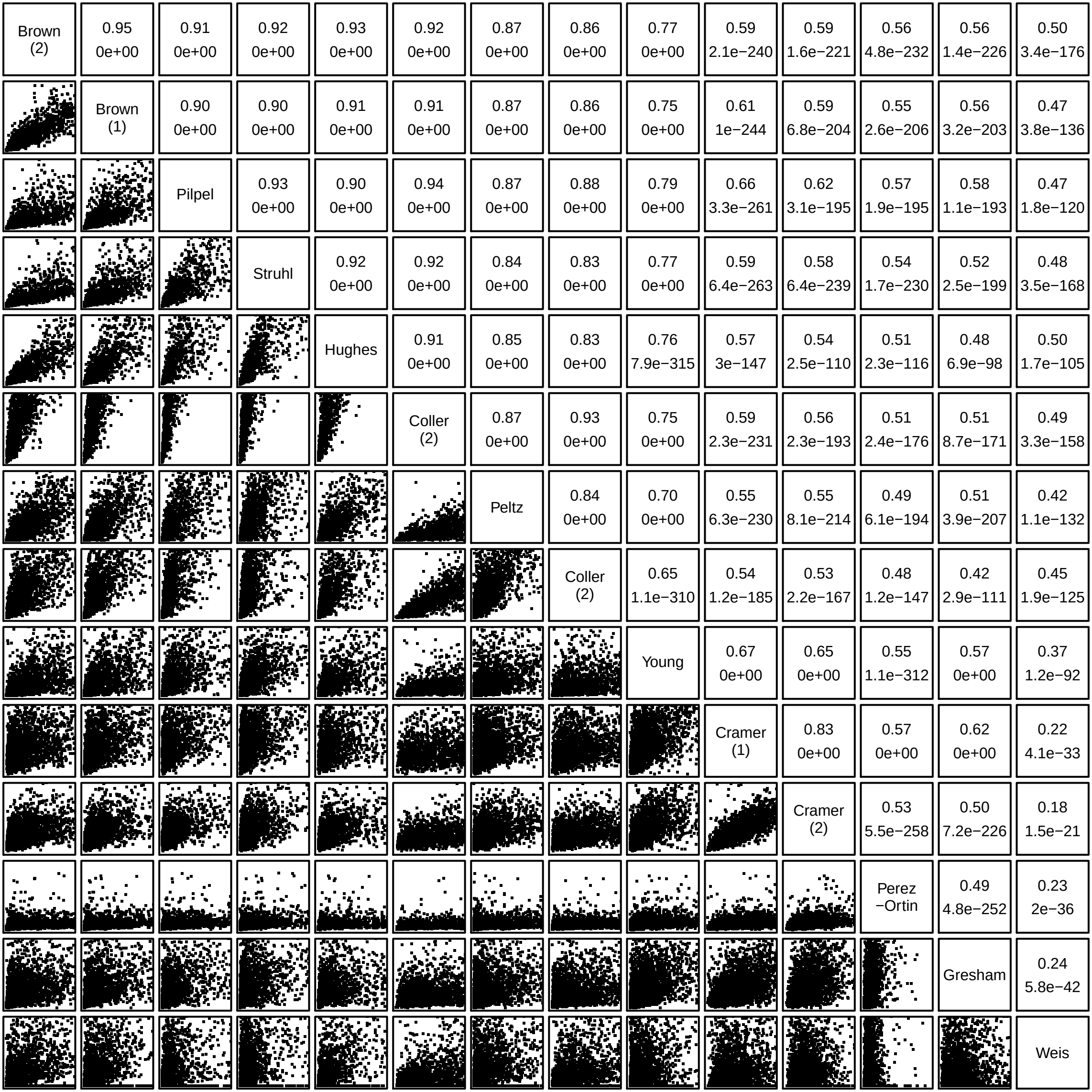
Brown (2)
0.95 0e+00
0.91 0e+00
0.92 0e+00
0.93 0e+00
0.92 0e+00
0.87 0e+00
0.86 0e+00
0.77 0e+00
0.59 2.1e−240
0.59 1.6e−221
0.56 4.8e−232
0.56 1.4e−226
0.50 3.4e−176
Brown (1)
0.90 0e+00
0.90 0e+00
0.91 0e+00
0.91 0e+00
0.87 0e+00
0.86 0e+00
0.75 0e+00
0.61 1e−244
0.59 6.8e−204
0.55 2.6e−206
0.56 3.2e−203
0.47 3.8e−136
Pilpel
0.93 0e+00
0.90 0e+00
0.94 0e+00
0.87 0e+00
0.88 0e+00
0.79 0e+00
0.66 3.3e−261
0.62 3.1e−195
0.57 1.9e−195
0.58 1.1e−193
0.47 1.8e−120
Struhl
0.92 0e+00
0.92 0e+00
0.84 0e+00
0.83 0e+00
0.77 0e+00
0.59 6.4e−263
0.58 6.4e−239
0.54 1.7e−230
0.52 2.5e−199
0.48 3.5e−168
Hughes
0.91 0e+00
0.85 0e+00
0.83 0e+00
0.76 7.9e−315
0.57 3e−147
0.54 2.5e−110
0.51 2.3e−116
0.48 6.9e−98
0.50 1.7e−105
Coller (2)
0.87 0e+00
0.93 0e+00
0.75 0e+00
0.59 2.3e−231
0.56 2.3e−193
0.51 2.4e−176
0.51 8.7e−171
0.49 3.3e−158
Peltz
0.84 0e+00
0.70 0e+00
0.55 6.3e−230
0.55 8.1e−214
0.49 6.1e−194
0.51 3.9e−207
0.42 1.1e−132
Coller (2)
0.65 1.1e−310
0.54 1.2e−185
0.53 2.2e−167
0.48 1.2e−147
0.42 2.9e−111
0.45 1.9e−125
Young
0.67 0e+00
0.65 0e+00
0.55 1.1e−312
0.57 0e+00
0.37 1.2e−92
Cramer (1)
0.83 0e+00
0.57 0e+00
0.62 0e+00
0.22 4.1e−33
Cramer (2)
0.53 5.5e−258
0.50 7.2e−226
0.18 1.5e−21
Perez −Ortin
0.49 4.8e−252
0.23 2e−36
Gresham
0.24 5.8e−42
Weis
