# Supplementary material for: Analysis of the association between codon optimality and mRNA stability in Schizosaccharomyces pombe
Source: BMC Genomics. 2016 Nov 8;17:895. doi: 10.1186/s12864-016-3237-6 (PMC5101800; doi:10.1186/s12864-016-3237-6)
Supplement: Additional file 4: Figure S4. — Comparison of mRNA synthesis rates in S. pombe. The pairwise scatterplots compare mRNA half-lives in seven datasets. The datasets are ordered as in Fig. 2d. The upper triangle panels show Spearman correlation coefficients (top) and P values (bottom). The axis range is from 0 to 1 RNA per minute. (PDF 434 kb) [file 12864_2016_3237_MOESM4_ESM.pdf]

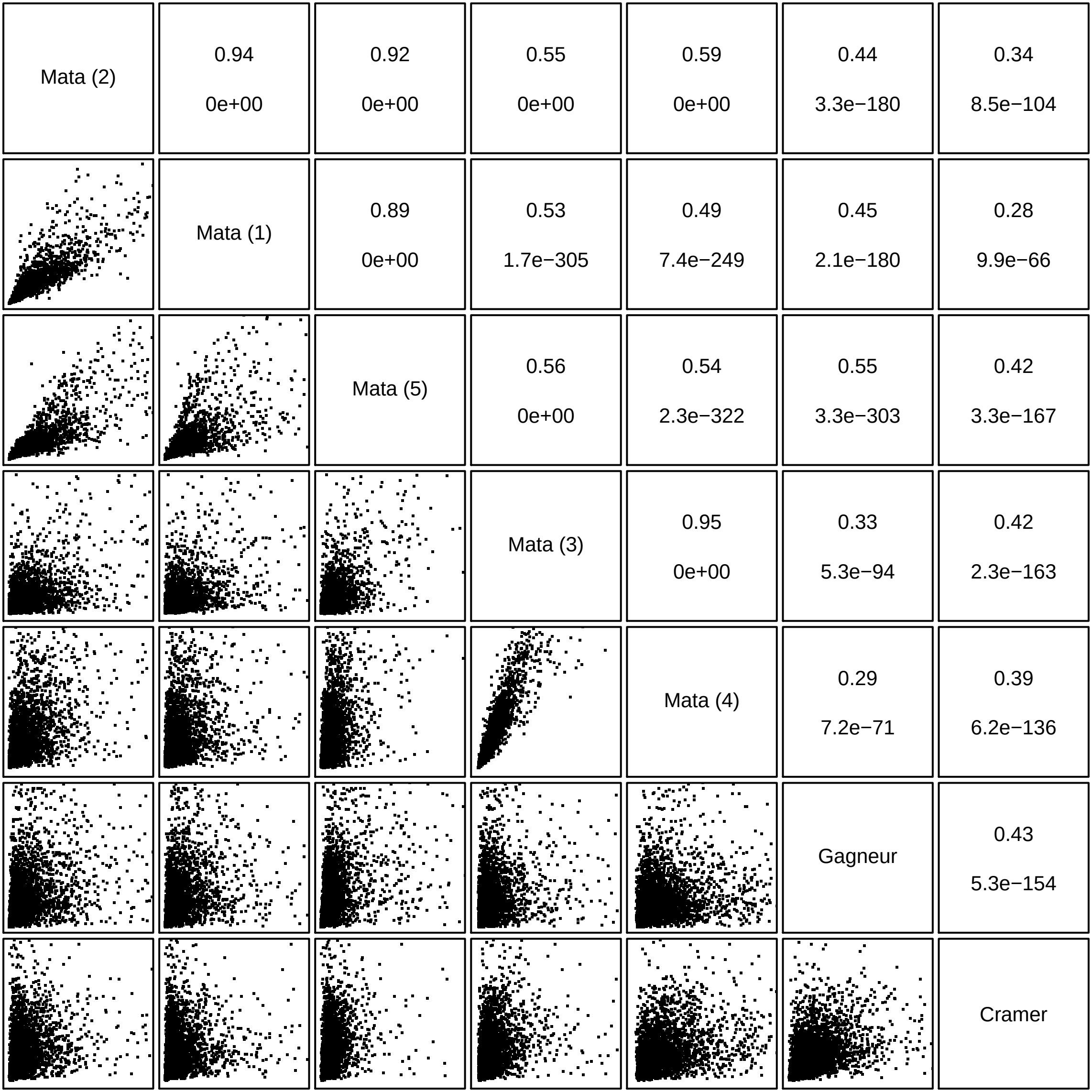
Mata (2)
0.94
0e+00
0.92
0e+00
0.55
0e+00
0.59
0e+00
0.44
3.3e−180
0.34
8.5e−104
Mata (1)
0.89
0e+00
0.53
1.7e−305
0.49
7.4e−249
0.45
2.1e−180
0.28
9.9e−66
Mata (5)
0.56
0e+00
0.54
2.3e−322
0.55
3.3e−303
0.42
3.3e−167
Mata (3)
0.95
0e+00
0.33
5.3e−94
0.42
2.3e−163
Mata (4)
0.29
7.2e−71
0.39
6.2e−136
Gagneur
0.43
5.3e−154
Cramer
