# Supplementary figures and images for: Analysis of the association between codon optimality and mRNA stability in Schizosaccharomyces pombe
Source: BMC Genomics. 2016 Nov 8;17:895. doi: 10.1186/s12864-016-3237-6 (PMC5101800; doi:10.1186/s12864-016-3237-6)

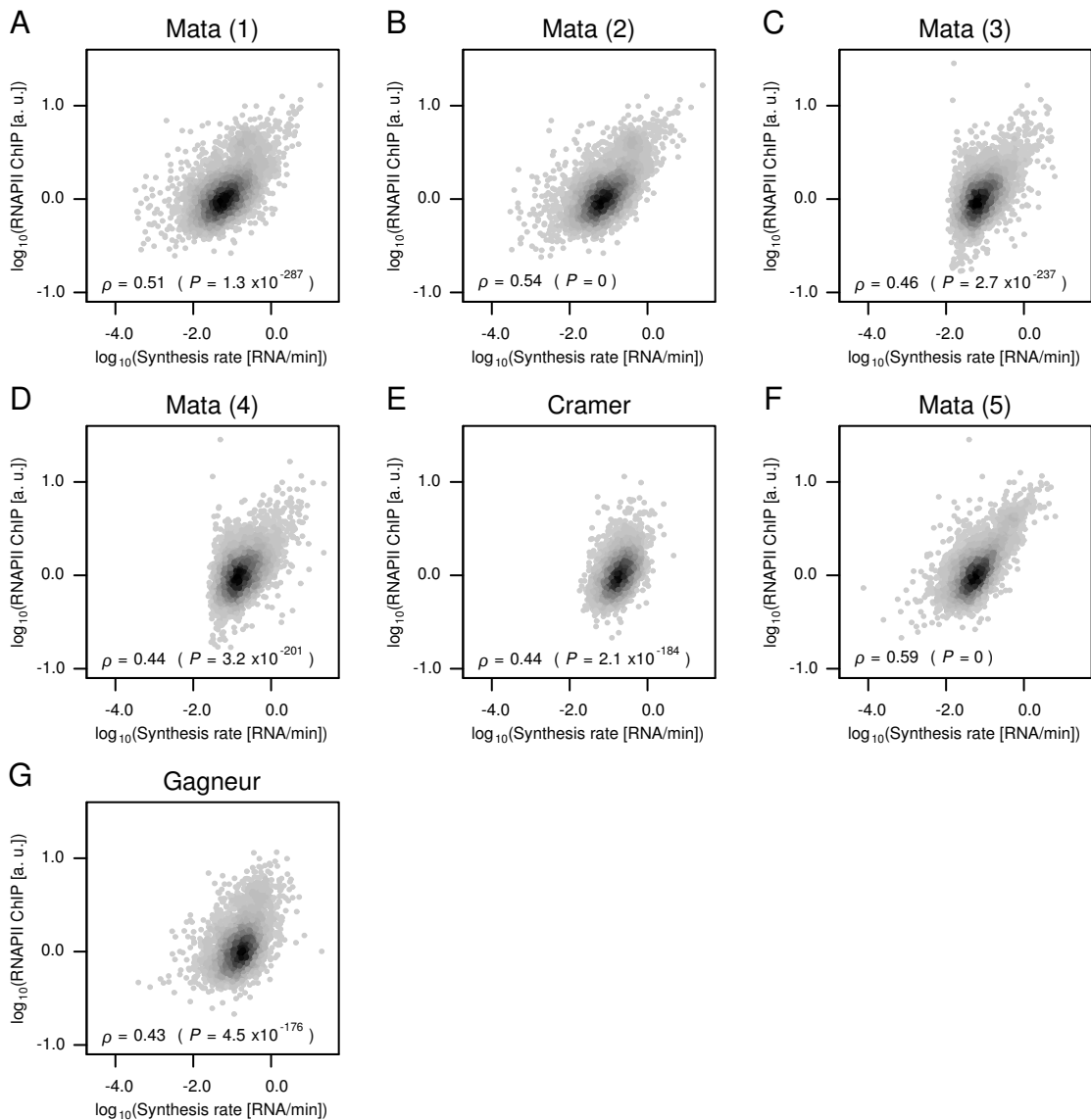

Supplement: Additional file 5: Figure S5. — Comparison of mRNA synthesis rates with ChIP intensity signals for RNAPII in S. pombe. (A-G) Scatterplot comparing mRNA synthesis rates [RNA/min] in seven datasets and RNAPII ChIP signals in an arbitrary unit (a. u.) obtained by Bahler and colleagues [55]. Spearman's ρ and P value are shown. (PDF 984 kb) [file 12864_2016_3237_MOESM5_ESM.pdf]

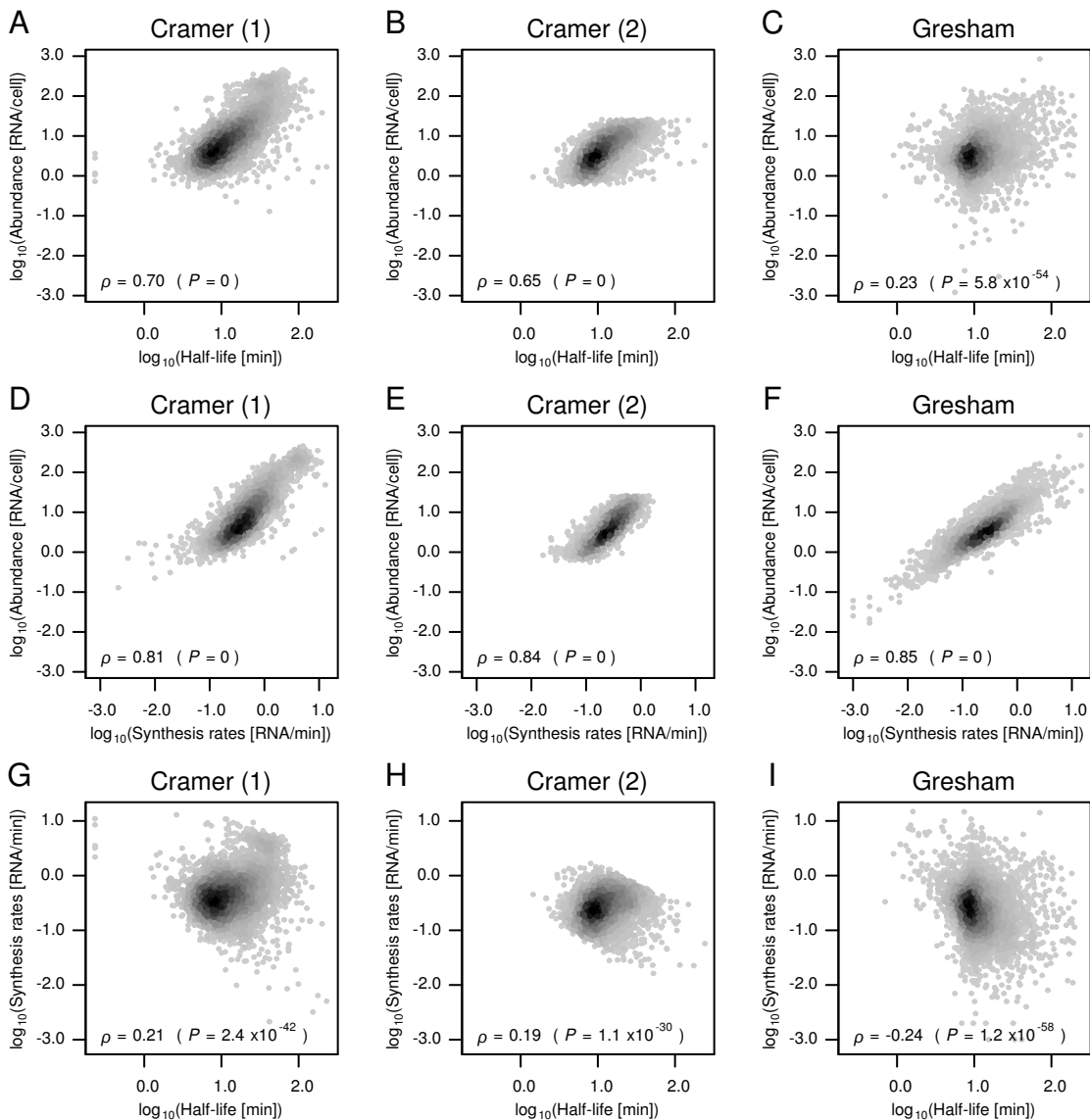

Supplement: Additional file 6: Figure S6. — Correlations between mRNA half-lives, synthesis rates, and abundance in S. cerevisiae. (A) Scatterplot comparing mRNA half-lives and abundance in the “Cramer (1)” data. Spearman's ρ and P value are shown. (B) Same as (A) but for the “Cramer (2)” data. (C) Same as (A) but for the “Gresham” data. (D) Scatterplot comparing mRNA synthesis rates and abundance in the “Cramer (1)” data. (E) Same as (D) but for the “Cramer (2)” data. (F) Same as (D) but for the “Gresham” data. (G) Scatterplot comparing mRNA half-lives and synthesis rates in the “Cramer (1)” data. (H) Same as (G) but for the “Cramer (2)” data. (I) Same as (G) but for the “Gresham” data. (PDF 1187 kb) [file 12864_2016_3237_MOESM6_ESM.pdf]

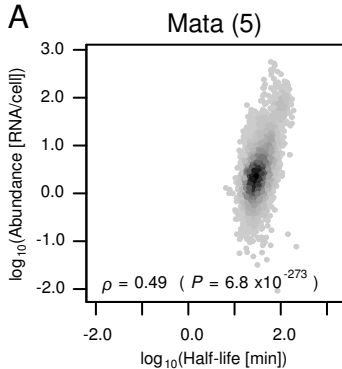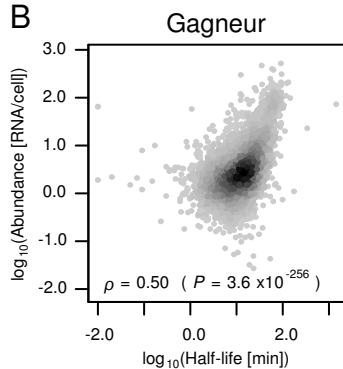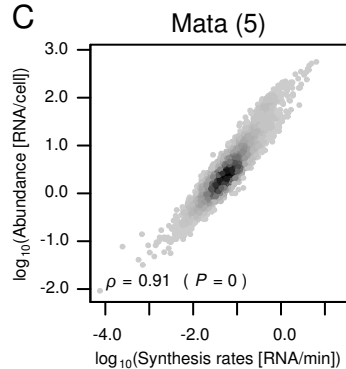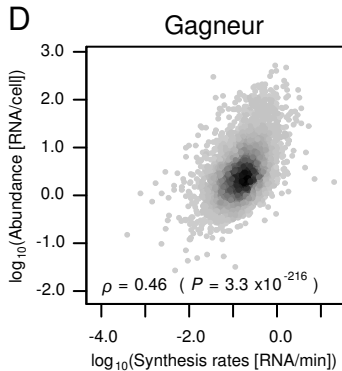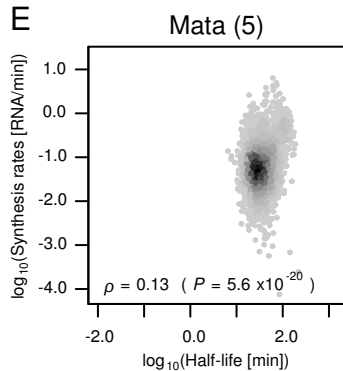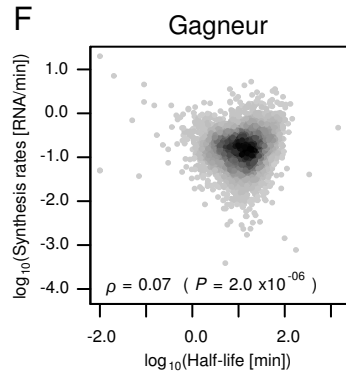

Supplement: Additional file 7: Figure S7. — Correlations between mRNA half-lives, synthesis rates, and abundance in S. pombe. (A) Scatterplot comparing mRNA half-lives in the “Mata (5)” data and mRNA abundance [RNA/cell] obtained by Bahler and colleagues [72]. Spearman's ρ and P value are shown. (B) Same as (A) but for mRNA half-lives in the “Gagneur” data. (C) Scatterplot comparing mRNA synthesis rates computed from the “Mata (5)” data and mRNA abundance as shown in (A). (D) Same as (C) but for mRNA synthesis rates in the “Gagneur” data. (E) Scatterplot comparing mRNA half-lives and synthesis rates in the “Mata (5)” data. (F) Same as (E) but for the “Gagneur” data. (PDF 818 kb) [file 12864_2016_3237_MOESM7_ESM.pdf]

A

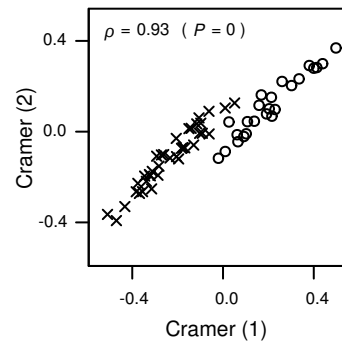

B

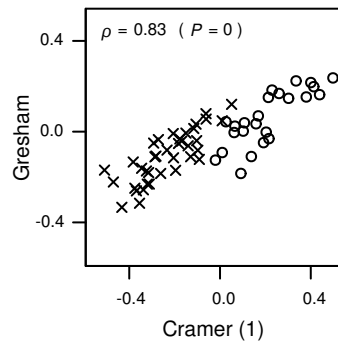

C

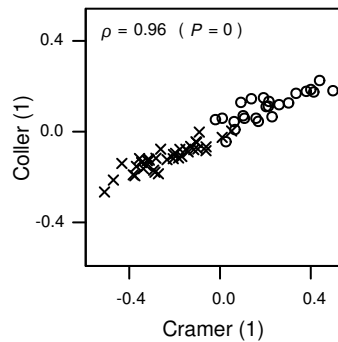

D

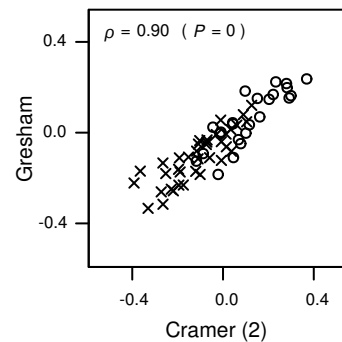

E

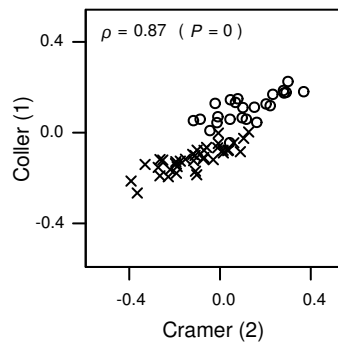

F

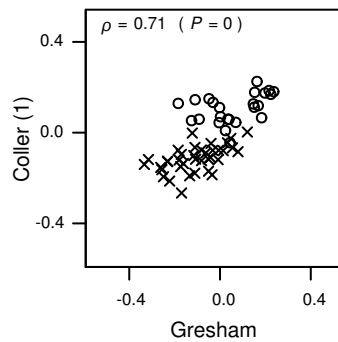

G

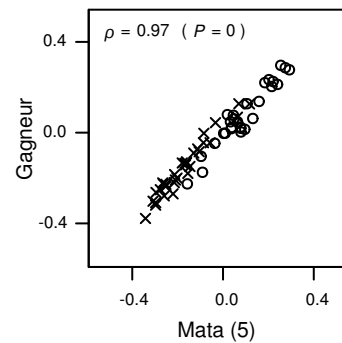

○ Optimal codons  
 × Non-optimal codons

Supplement: Additional file 8: Figure S8. — Correlations between the CSC values obtained from different RNA kinetic datasets in S. cerevisiae (“Cramer (1),” “Cramer (2),” “Gresham,” and “Coller (1)”) (A-F) and S. pombe (“Mata (5)” and “Gagneur”) (G). Spearman's ρ and P value are shown. The circles and cross signs represent optimal and non-optimal codons, respectively. (PDF 38 kb) [file 12864_2016_3237_MOESM8_ESM.pdf]

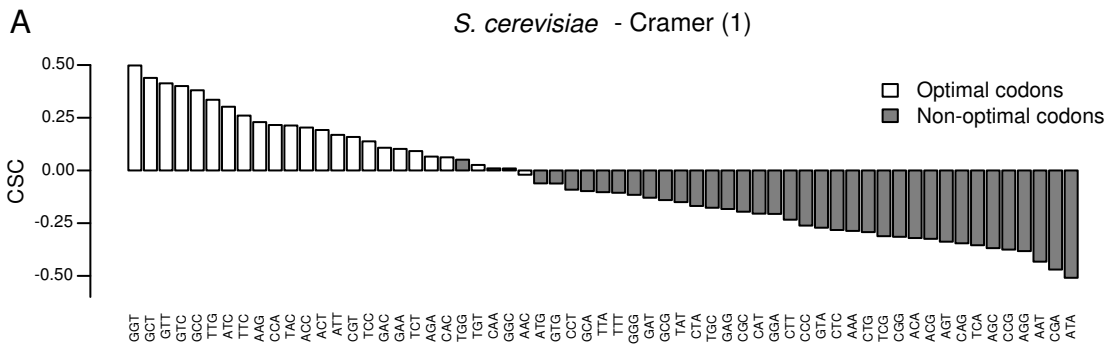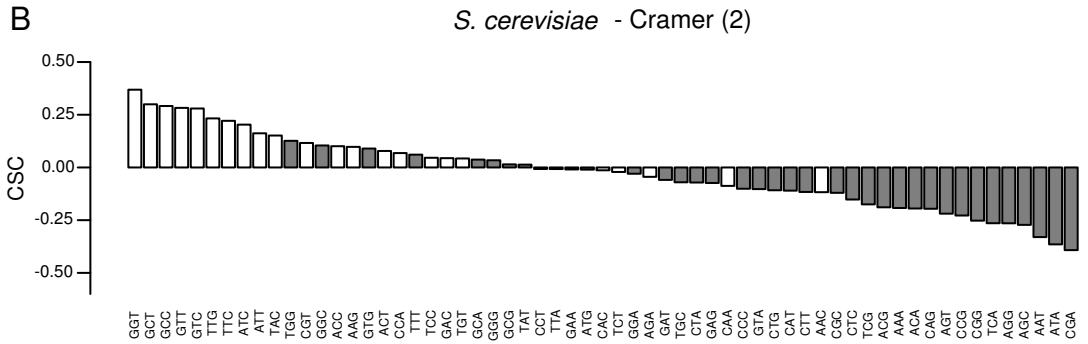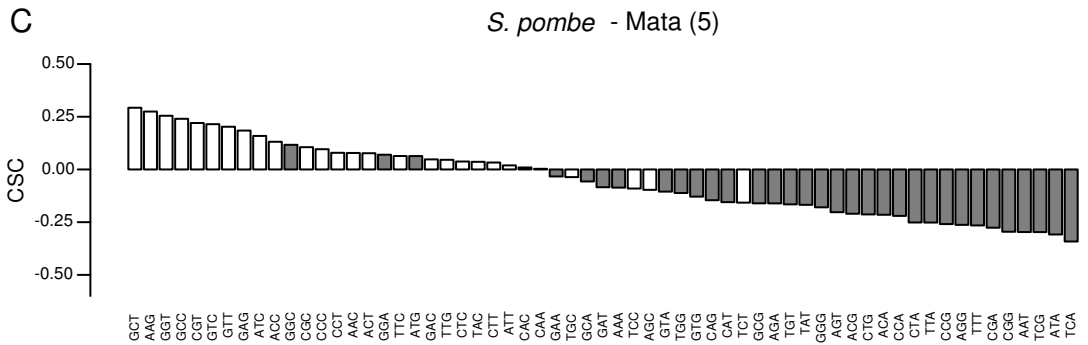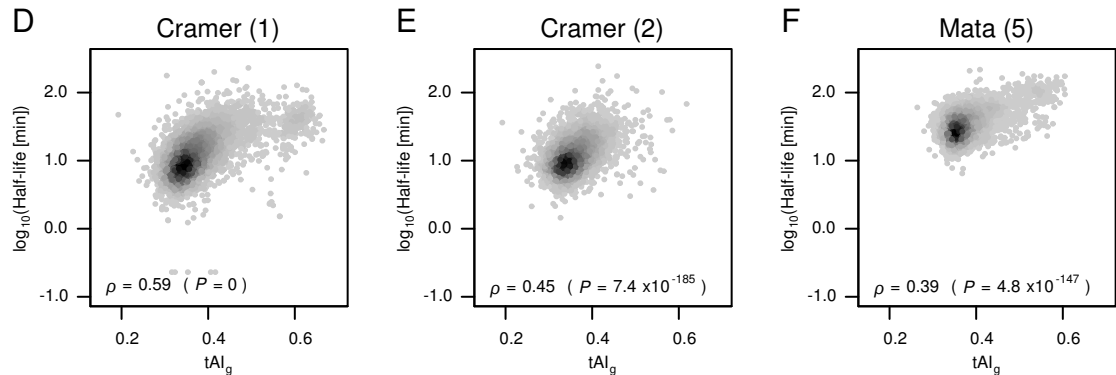

Supplement: Additional file 9: Figure S9. — Codon optimality and mRNA half-lives are significantly associated in S. cerevisiae and S. pombe. (A) The CSC plotted for each codon based on S. cerevisiae mRNA half-lives in the “Cramer (1)” dataset. The white and gray bars represent optimal and non-optimal codons, respectively. The classification of codon optimality is based on the S. cerevisiae cTE. (B) Same as (A) but for the “Cramer (2)” dataset. (C) The CSC plotted for each codon based on S. pombe mRNA half-lives in the “Mata (5)” dataset. The classification of codon optimality is based on the S. pombe cTE. (D) Scatterplot comparing tAIg and mRNA half-lives in the “Cramer (1)” dataset in S. cerevisiae. Spearman's ρ and P value are shown. (E) Same as (D) but for the “Cramer (2)” data. (F) Same as (D) but for the “Mata (5)” dataset in S. pombe. (PDF 427 kb) [file 12864_2016_3237_MOESM9_ESM.pdf]

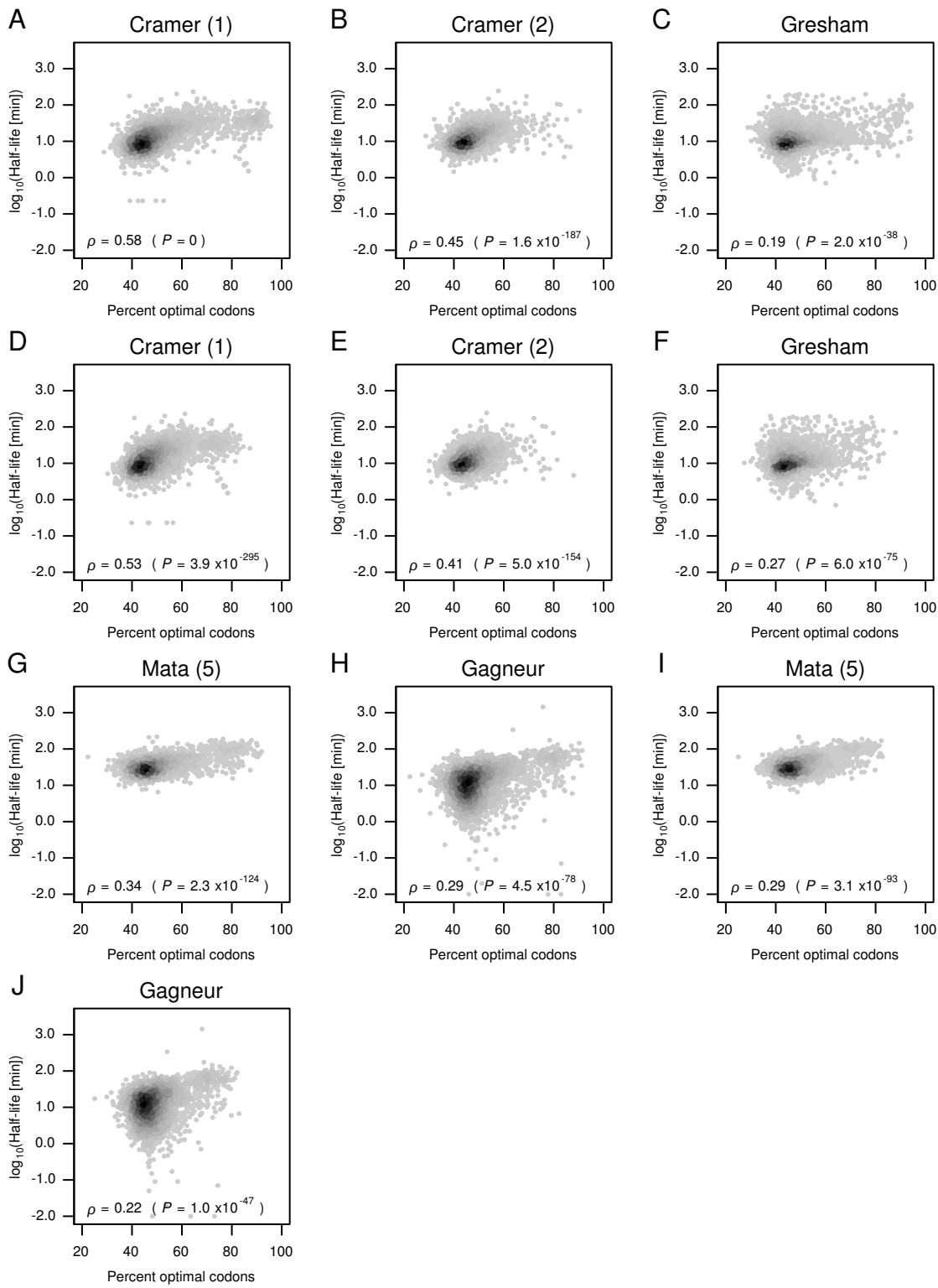

Supplement: Additional file 10: Figure S10. — Correlations between optimal codon content and mRNA half-lives in S. cerevisiae and S. pombe. (A) Scatterplot comparing optimal codon content based on the cTE classification and mRNA half-lives in the “Cramer (1)” data in S. cerevisiae. Spearman's ρ and P value are shown. (B) Same as (A) but for the “Cramer (2)” data. (C) Same as (A) but for the “Gresham” data. (D) Same as (A) but based on the nTE classification. (E) Same as (D) but for the “Cramer (2)” data. (F) Same as (D) but for the “Gresham” data. (G) Same as (A) but for the “Mata (5)” data in S. pombe. (H) Same as (G) but for the “Gagneur” data. (I) Same as (G) but based on the nTE classification. (J) Same as (I) but for the “Gagneur” data. (PDF 1341 kb) [file 12864_2016_3237_MOESM10_ESM.pdf]

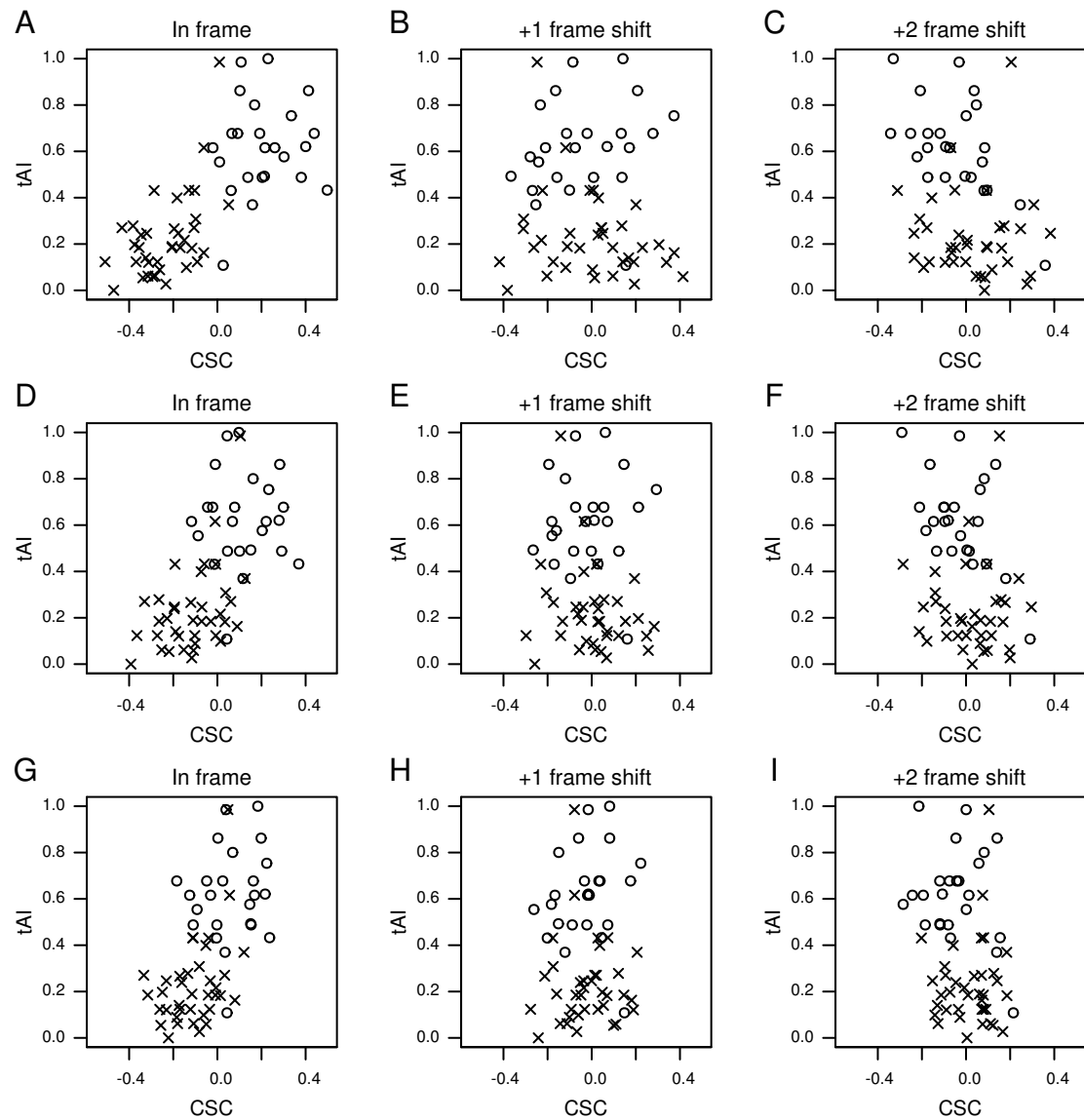

Supplement: Additional file 11: Figure S11. — Introduction of +1 and +2 frameshifts eliminates the positive correlation between codon optimality and the CSC in S. cerevisiae. (A) Scatterplot comparing the tAI values and the CSC based on the “Cramer (1)” data (ρ = 0.76, P = 1.7 × 10^−12^). The circles and cross signs represent optimal and non-optimal codons, respectively. (B) Same as (A) but upon introduction of +1 frameshifts (ρ = −0.12, P = 0.37). (C) Same as (A) but upon introduction of +2 frameshifts (ρ = −0.34, P = 7.7 × 10^−3^). (D) Same as (A) but based on the “Cramer (2)” data (ρ = 0.62, P = 9.3 × 10^−8^). (E) Same as (B) but based on the “Cramer (2)” data (ρ = −0.16, P = 0.21). (F) Same as (C) but based on the “Cramer (2)” data (ρ = −0.29, P = 0.02). (G) Same as (A) but based on the “Gresham” data (ρ = 0.58, P = 1.0 × 10^−6^). (H) Same as (B) but based on the “Gresham” data (ρ = 0.03, P = 0.76). (I) Same as (C) but based on the “Gresham” data (ρ = −0.23, P = 0.07). (PDF 29 kb) [file 12864_2016_3237_MOESM11_ESM.pdf]

A

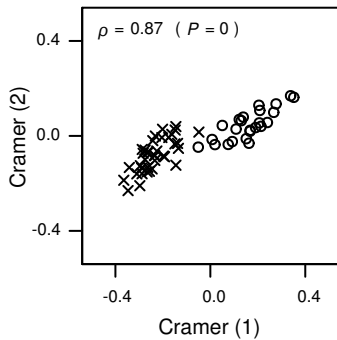

B

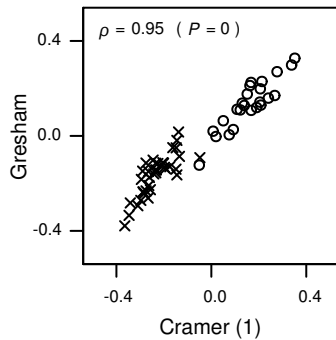

C

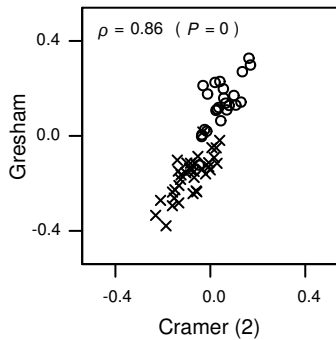

D

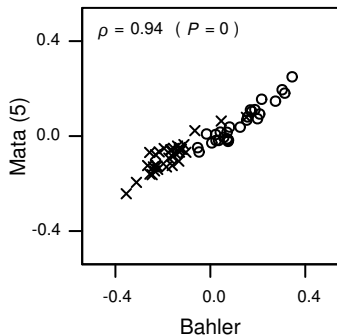

E

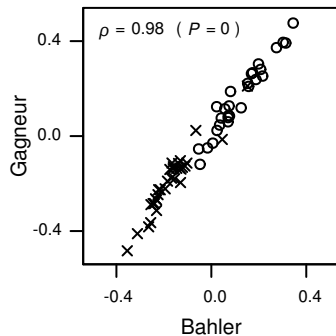

F

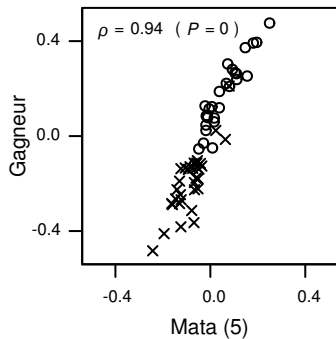

○ Optimal codons  
 × Non-optimal codons

Supplement: Additional file 14: Figure S13. — Correlations between the CPC values obtained from different RNA kinetic datasets in S. cerevisiae (“Cramer (1),” “Cramer (2),” and “Gresham”) (A-C) and S. pombe (“Mata (5)” and “Gagneur”) as well as those obtained from the RNAPII ChIP data in S. pombe (“Bahler”) (D-F) [55]. Spearman's ρ and P value are shown. The circles and cross signs represent optimal and non-optimal codons, respectively. (PDF 36 kb) [file 12864_2016_3237_MOESM14_ESM.pdf]

**A****Cramer (1)**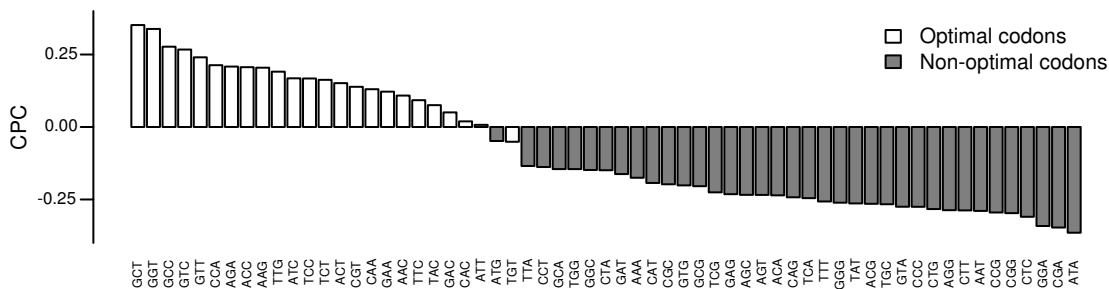**B****Cramer (2)**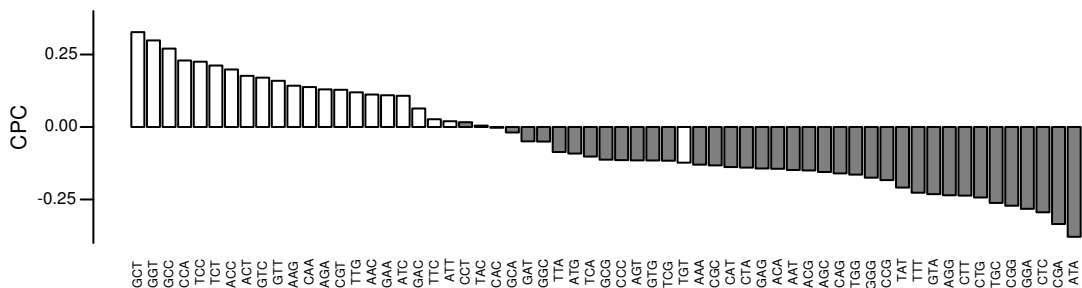**C****Cramer (1)**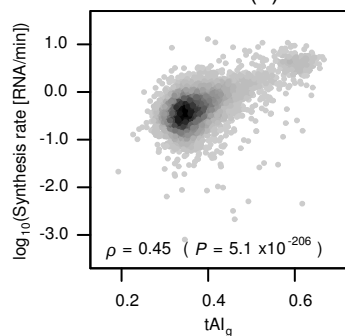**D****Cramer (2)**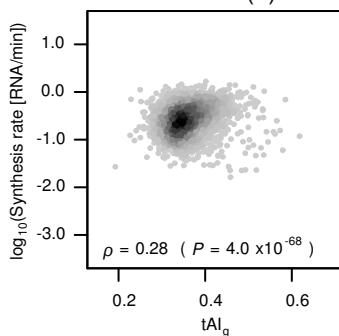

Supplement: Additional file 15: Figure S14. — Codon optimality and mRNA synthesis rates are significantly associated in S. cerevisiae. (A) The CPC plotted for each codon based on mRNA synthesis rates in the “Cramer (1)” dataset. The white and gray bars represent optimal and non-optimal codons, respectively. The classification of codon optimality is based on the S. cerevisiae cTE. (B) Same as (A) but based on the “Cramer (2)” dataset. (C) Scatterplot comparing tAIg and mRNA synthesis rates in the “Cramer (1)” dataset. Spearman's ρ and P value are shown. (D) Same as (C) but for the “Cramer (2)” dataset. (PDF 275 kb) [file 12864_2016_3237_MOESM15_ESM.pdf]

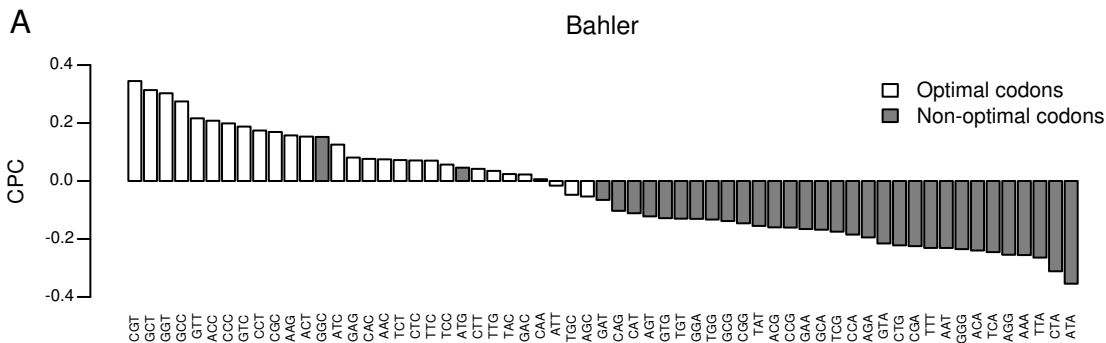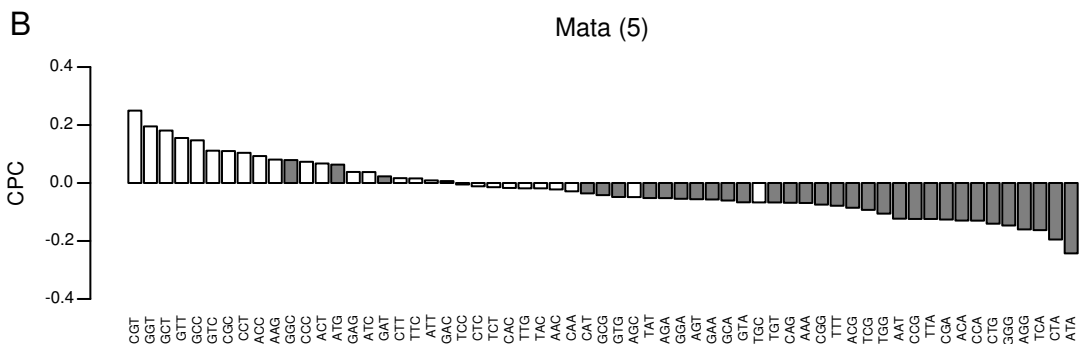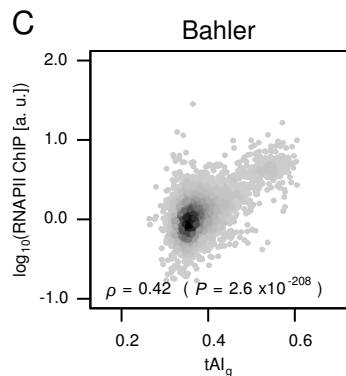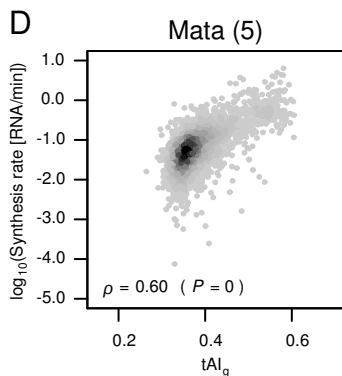

Supplement: Additional file 16: Figure S15. — Codon optimality and mRNA synthesis rates are significantly associated in S. pombe. (A) The CPC plotted for each codon based on the RNAPII ChIP signals by Bahler and colleagues [55]. The white and gray bars represent optimal and non-optimal codons, respectively. The classification of codon optimality is based on the S. pombe cTE. (B) Same as (A) but for the CPC based on mRNA synthesis rates in the “Mata (5)” dataset. (C) Scatterplot comparing tAIg and RNAPII ChIP signals by Bahler and colleagues. Spearman's ρ and P value are shown. (D) Same as (C) but for mRNA synthesis rates in the “Mata (5)” dataset. (PDF 323 kb) [file 12864_2016_3237_MOESM16_ESM.pdf]

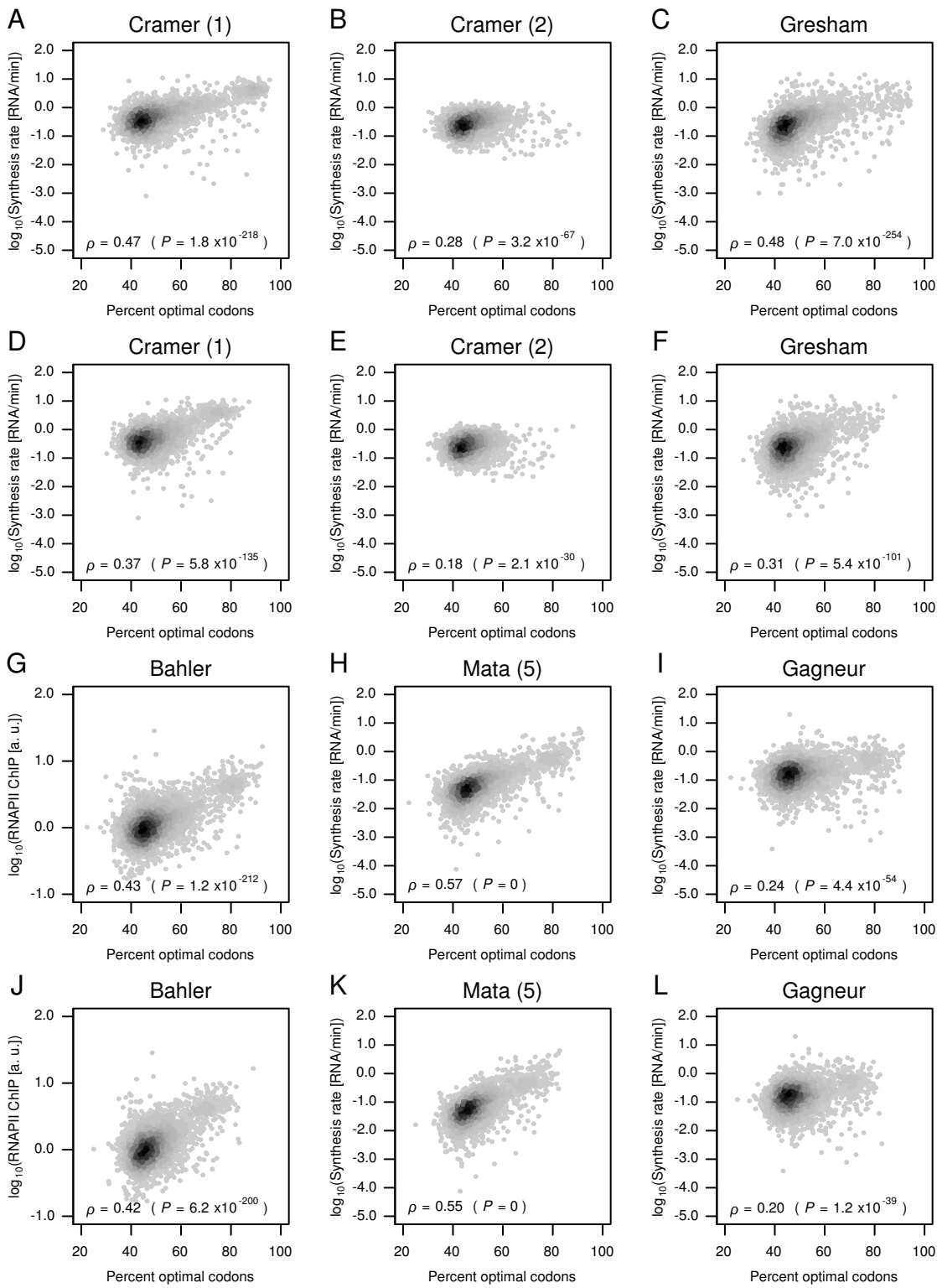

Supplement: Additional file 17: Figure S16. — Correlations between optimal codon content and mRNA synthesis rates in S. cerevisiae and S. pombe. (A) Scatterplot comparing optimal codon content based on the cTE classification and mRNA synthesis rates in the “Cramer (1)” data in S. cerevisiae. Spearman's ρ and P value are shown. (B) Same as (A) but for the “Cramer (2)” data. (C) Same as (A) but for the “Gresham” data. (D) Same as (A) but based on the nTE classification. (E) Same as (D) but for the “Cramer (2)” data. (F) Same as (D) but for the “Gresham” data. (G) Same as (A) but for the S. pombe RNAPII ChIP data by Bahler and colleagues. (H) Same as (A) but for the “Mata (5)” data in S. pombe. (I) Same as (G) but for the “Gagneur” data. (J) Same as (G) but based on the nTE classification. (K) Same as (J) but for the “Mata (5)” data. (L) Same as (J) but for the “Gagneur” data. (PDF 1653 kb) [file 12864_2016_3237_MOESM17_ESM.pdf]

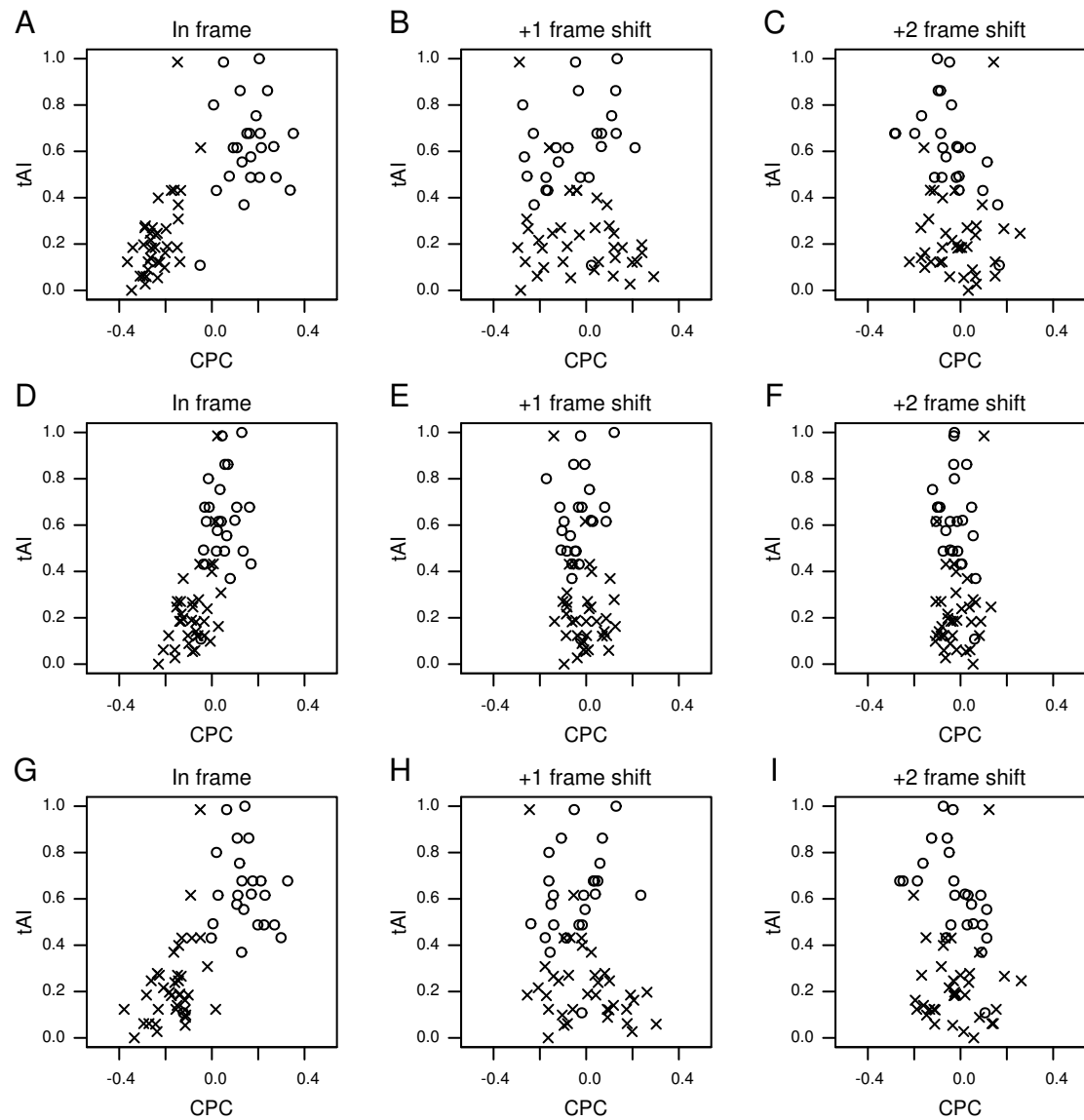

Supplement: Additional file 18: Figure S17. — Introduction of +1 and +2 frameshifts eliminates the positive correlation between codon optimality and the CPC in S. cerevisiae. (A) Scatterplot comparing the tAI values and the CPC based on the “Cramer (1)” data (ρ = 0.79, P = 6.6 × 10^−14^). The circles and cross signs represent optimal and non-optimal codons, respectively. (B) Same as (A) but upon introduction of +1 frameshifts (ρ = −0.10, P = 0.44). (C) Same as (A) but upon introduction of +2 frameshifts (ρ = −0.28, P = 0.03). (D) Same as (A) but based on the “Cramer (2)” data (ρ = 0.74, P = 1.4 × 10^−11^). (E) Same as (B) but based on the “Cramer (2)” data (ρ = −0.16, P = 0.22). (F) Same as C but based on the “Cramer (2)” data (ρ = −0.05, P = 0.72). (G) Same as (A) but based on the “Gresham” data (ρ = 0.75, P = 3.4 × 10^−12^). (H) Same as (B) but based on the “Gresham” data (ρ = −0.17, P = 0.18). (I) Same as (C) but based on the “Gresham” data (ρ = −0.15, P = 0.26). (PDF 29 kb) [file 12864_2016_3237_MOESM18_ESM.pdf]

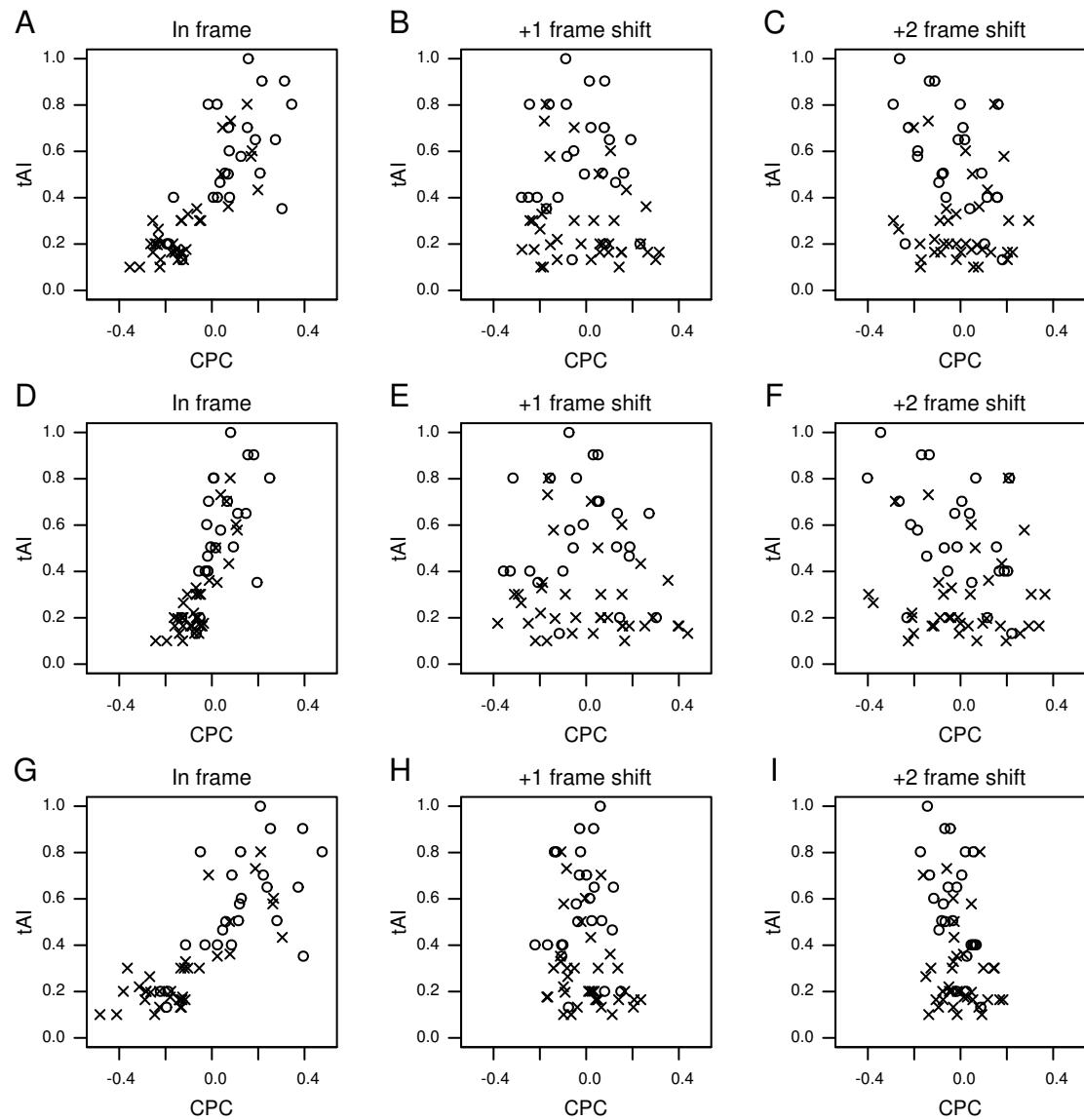

Supplement: Additional file 19: Figure S18. — Introduction of +1 and +2 frameshifts eliminates the positive correlation between codon optimality and the CPC in S. pombe. (A) Scatterplot comparing the tAI values and the CPC based on the RNAPII ChIP signals by Bahler and colleagues (ρ = 0.80, P = 5.5 × 10^−15^). The circles and cross signs represent optimal and non-optimal codons, respectively. (B) Same as (A) but upon introduction of +1 frameshifts (ρ = −0.14, P = 0.29). (C) Same as (A) but upon introduction of +2 frameshifts (ρ = −0.22, P = 0.09). (D) Same as (A) but for the CPC based on mRNA synthesis rates in the “Mata (5)” data (ρ = 0.83, P = 1.4 × 10^−16^). (E) Same as (B) but for the CPC based on mRNA synthesis rates in the “Mata (5)” data (ρ = −0.15, P = 0.25). (F) Same as (C) but for the CPC based on mRNA synthesis rates in the “Mata (5)” data (ρ = −0.19, P = 0.13). (G) Same as (D) but based on the “Gagneur” data (ρ = 0.82, P = 4.2 × 10^−16^). (H) Same as (E) but based on the “Gagneur” data (ρ = −0.20, P = 0.11). (I) Same as (F) but based on the “Gagneur” data (ρ = −0.28, P = 0.03). (PDF 29 kb) [file 12864_2016_3237_MOESM19_ESM.pdf]
