# Supplementary material for: Analysis of the association between codon optimality and mRNA stability in Schizosaccharomyces pombe
Source: BMC Genomics. 2016 Nov 8;17:895. doi: 10.1186/s12864-016-3237-6 (PMC5101800; doi:10.1186/s12864-016-3237-6)
Supplement: Additional file 12: Figure S12. — Introduction of +1 and +2 frameshifts eliminates the positive correlation between codon optimality and the CSC in S. pombe. (A) Scatterplot comparing the tAI values and the CSC based on the “Mata (5)” data (ρ = 0.85, P = 7.6 × 10^−18^). The circles and cross signs represent optimal and non-optimal codons, respectively. (B) Same as (A) but upon introduction of +1 frameshifts (ρ = −0.16, P = 0.22). (C) Same as (A) but upon introduction of +2 frameshifts (ρ = −0.30, P = 0.02). (D) Same as (A) but based on the “Gagneur” data (ρ = 0.81, P = 4.6 × 10^−15^). (E) Same as (B) but based on the “Gagneur” data (ρ = −0.16, P = 0.02). (F) Same as (C) but based on the “Gagneur” data (ρ = −0.37, P = 3.8 × 10^−3^). (PDF 24 kb) [file 12864_2016_3237_MOESM12_ESM.pdf]

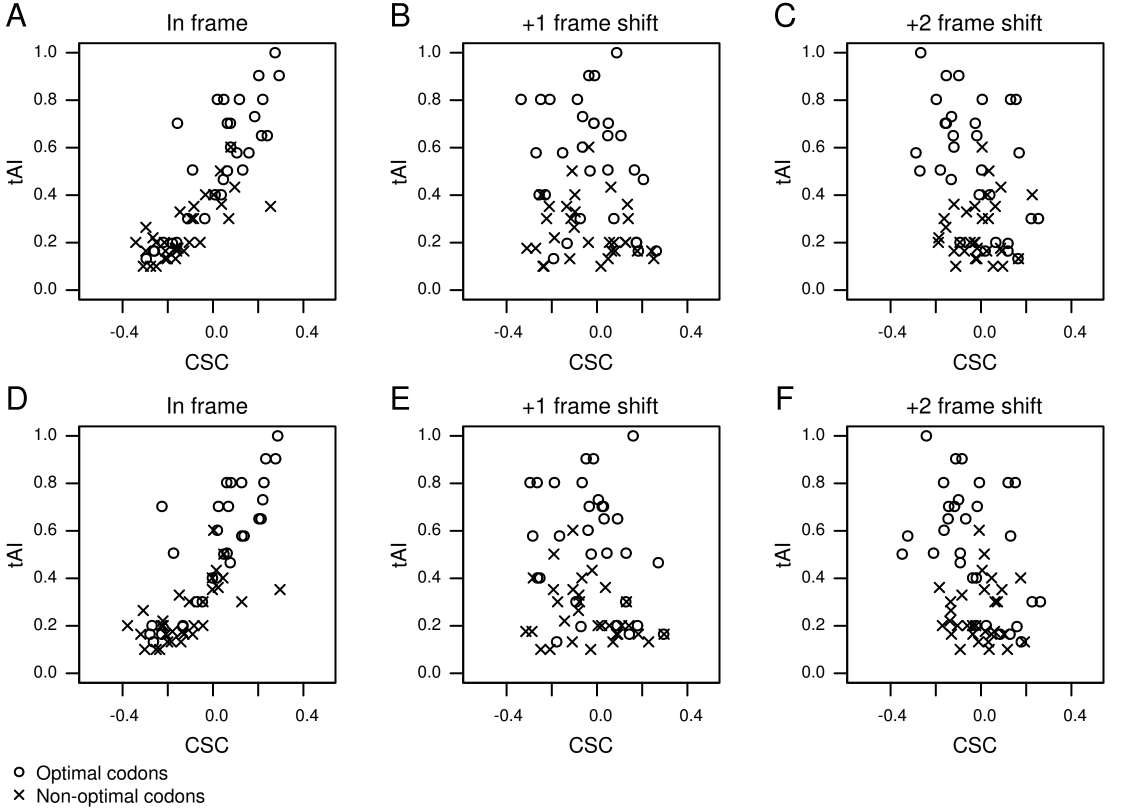
A
In frame
B
+1 frame shift
C
+2 frame shift
D
In frame
E
+1 frame shift
F
+2 frame shift
tAI
CSC
1.0
0.8
0.6
0.4
0.2
0.0
-0.4
0.0
0.4
Optimal codons
Non-optimal codons
